# Association of COX-inhibitors with cancer patients’ survival under chemotherapy and radiotherapy regimens: a real-world data retrospective cohort analysis

**DOI:** 10.3389/fonc.2024.1433497

**Published:** 2024-09-13

**Authors:** Lucas E. Flausino, Isabella N. Ferreira, Wen-Jan Tuan, Maria Del Pilar Estevez-Diz, Roger Chammas

**Affiliations:** ^1^ Center for Translational Research in Oncology, Instituto do Câncer do Estado de São Paulo, Faculdade de Medicina da Universidade de São Paulo, Comprehensive Center for Precision Oncology, Universidade de São Paulo, São Paulo, SP, Brazil; ^2^ Department of Family and Community Medicine, and Public Health Sciences, Penn State College of Medicine, Hershey, PA, United States; ^3^ Division of Clinical Oncology, Instituto do Câncer do Estado de São Paulo, Hospital das Clínicas da Faculdade de Medicina da Universidade de São Paulo, Comprehensive Center for Precision Oncology, Universidade de São Paulo, São Paulo, SP, Brazil

**Keywords:** COX-inhibitors, non-steroidal anti-inflammatory drugs, cancer survival, chemotherapy, radiotherapy, multiarm, multistage, platform

## Abstract

**Introduction:**

We conducted an extensive, sex-oriented real-world data analysis to explore the impact and safety of non-steroidal anti-inflammatory drugs (NSAIDs) and selective COX-2 inhibitors (coxibs) on cancer treatment outcomes. This is particularly relevant given the role of the COX-2/PGE2 pathway in tumor cell resistance to chemotherapy and radiotherapy.

**Methods:**

The study applied a retrospective cohort design utilizing the TriNetX research database consisting of patients receiving cancer treatment in 2008-2022. The treated cohorts included patients who were prescribed with coxibs, aspirin or ibuprofen, while individuals in the control cohort did not receive these medicines during their cancer treatment. A 1:1 propensity score matching technique was used to balance the baseline characteristics in the treated and control cohorts. Then, Cox proportional hazards regression and logistic regression were applied to assess the mortality and morbidity risks among patient cohorts in a 5-year follow-up period.

**Results:**

Use of coxibs (HR, 0.825; 95% CI 0.792-0.859 in females and HR, 0.884; 95% CI 0.848-0.921 in males) and ibuprofen (HR, 0.924; 95% CI 0.903-0.945 in females and HR, 0.940; 95% CI 0.917-0.963 in males) were associated with improved survival. Female cancer patients receiving aspirin presented increased mortality (HR, 1.078; 95% CI 1.060-1.097), while male cancer patients also had improved survival when receiving aspirin (HR, 0.966; 95% CI 0.951-0.980). Cancer subtype specific analysis suggests coxibs and ibuprofen correlated with survival, though ibuprofen and aspirin increased emergency department visits’ risk. Secondary analyses, despite limited by small cohort sizes, suggest that COX inhibition post-cancer diagnosis may benefit patients with specific cancer subtypes.

**Discussion:**

Selective COX-2 inhibition significantly reduced mortality and emergency department visit rates. Further clinical trials are needed to determine the optimal conditions for indication of coxibs as anti-inflammatory adjuvants in cancer treatment.

## Graphical Abstract

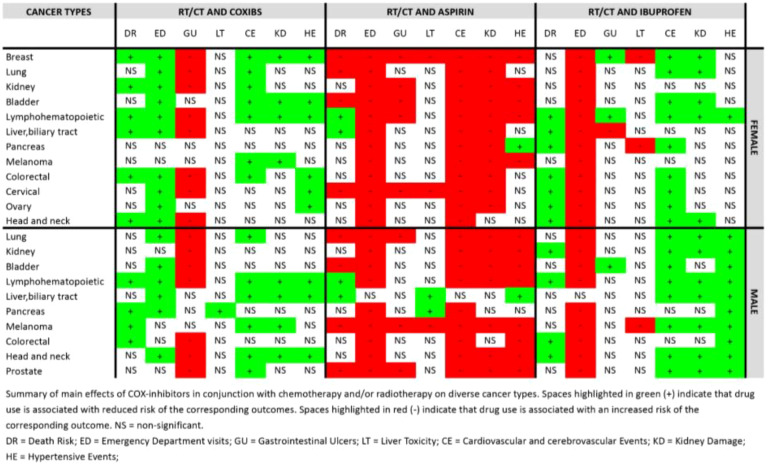

## 1 Introduction

Cancer’s ability to adapt and develop treatment resistance has long been implicated in disease progression and cancer mortality. Different mechanisms can contribute to this phenomenon, including quiescent stem-cell populations, epigenetic reprogramming, sub-lethal caspase oncogenic activity, and dying cells’ release of oncogenic molecules that remodel the microenvironment to support tumor growth, angiogenesis, and immune evasion (1–4).

Recent research has indicated that lipid mediators and altered lipid metabolism play a significant role in tumor cell repopulation and pro-oncogenic effects (5, 6). Of note, phosphatidylcholine (PtdCHo) metabolism is increased in tumor cells (7–10), generating lipid factors that act as signaling molecules to cell survival, proliferation, and immune modulation, contributing to treatment resistance (4, 6, 11–13).

The high concentration of these mediators, notably arachidonic acid (AA), upregulates the cyclooxygenase 2 (COX-2) pathway and its terminal product prostaglandin E2 (PGE2) (5, 6), promoting chronic inflammation, and stimulating the growth of surviving tumor cells, epithelial-mesenchymal transition (EMT), and the activity of immunosuppressive cells, as well as inhibiting antitumor response (4–6). Upon chemotherapy and radiotherapy, there is massive cell death, generation of platelet-activation factor receptor (PAFR) ligands, and production of reactive oxygen species (ROS) that can induce lipid peroxidation and ferroptosis, contributing to COX-2/PGE2 pathway activation, which results in chemo and radioresistance (4, 5, 14–17).

In this context, the COX-2/PGE-2 pathway is an appealing target once its blockage can have protective effects by reducing cancer progression and immune evasion as well as sensitizing tumor cells to apoptogenic therapies, such as chemotherapy and radiotherapy (16, 18–21). Indeed, the use of COX-2 inhibitors combined with conventional chemotherapy improved anti-tumor activity and patient tolerability (22, 23) and demonstrated antiangiogenic (24) and pro-apoptotic (25, 26) functions. Combined with radiotherapy, this strategy increased tumor cells’ susceptibility to radiation effects, inhibited cell proliferation, stimulated apoptosis, and decreased radiation toxicity to healthy tissues (23, 27).

Not only can COX-2 inhibitors interfere with cancer-dependent COX-2/PGE-2 proliferation and resistance, but they can also have anticancer activity via COX-2-independent mechanisms. As an example, Celecoxib, a specific COX-2 inhibitor, was found to induce apoptosis via p53 activation (28) and mitochondrial mechanisms (29), sensitize tumors to radiotherapy via AKT/mTOR (30), and interfere with tumors ability to adapt to an acidic microenvironment (31, 32). Other COX-2 inhibitors can induce cell cycle arrest, apoptosis, necrosis and interfere with the PIK3 pathway to reduce cell migration and proliferation (33, 34).

Recent research indicates that COX-2 inhibition can also have synergistic effects with tyrosine kinase inhibition (35–38), immunotherapy (39–41), and anti-angiogenic therapy (42). Moreover, COX-2 inhibitors can potentially interfere with tumor-suppressive and oncogenic microRNAs (miRNAs), culminating in an anticancer response (43).

Despite the current progress on COX-2 inhibitors as anticancer agents (23, 28) and the potential effect of these drugs and nonsteroidal anti-inflammatory drugs (NSAIDs) in reducing the risk of cancer incidence, progression, recurrence, and promoting higher survival rates in different cancer types (44–53), information on whether this strategy might be beneficial for patients in use of apoptogenic therapies remains inconclusive (54–60).

Additionally, the lack of information on the safety profile of the association between COX-inhibitors and current anticancer therapies is still a major concern for implementing this approach. Because of COX-inhibitors renal, gastrointestinal, and cardiovascular adverse effects (61, 62), its clinical implementation needs to be carefully evaluated. An association with a chemotherapeutic such as doxorubicin, for example, could potentially enhance its cardiovascular effects (23).

Therefore, to understand the real impact of COX-inhibition when associated with apoptogenic therapies, we performed an extensive analysis using real-world data of patients with various cancer types to evaluate the benefits and the safety profile of this approach. Using TriNetx Research Network, a database of electronic health records (EHR), we assessed the association between NSAIDS, such as aspirin and ibuprofen, and selective COX-2 inhibitors, such as coxibs, with mortality of cancer patients under chemotherapy and/or, radiotherapy. Our analysis included patients with bladder, breast, colorectal, cervical, head and neck, kidney, lymphohematopoietic, liver and biliary tract, lung, melanoma, ovarian, pancreatic, and prostate cancers. Additionally, we investigated the association of these medications with patient emergency visits risk and potential toxic effects (61, 62).

## 2 Methods

### 2.1 Study design and data source

This was a retrospective cohort study utilizing EHR of 83 healthcare organizations from the TriNetX Research Network, a large database consisting of de-identified, aggregated EHR data (demographics, diagnoses, procedures, medications, and laboratory tests) of more than 115 million patients (Cambridge, MA). All the data queries were performed in the TriNetX online portal, and the results contained only aggregated counts and statistical summaries. Because there was no patient-level identifiable data involved or accessed in the analysis, this research was determined to be exempt from the Institutional Review Board oversight.

**Figure 1:**
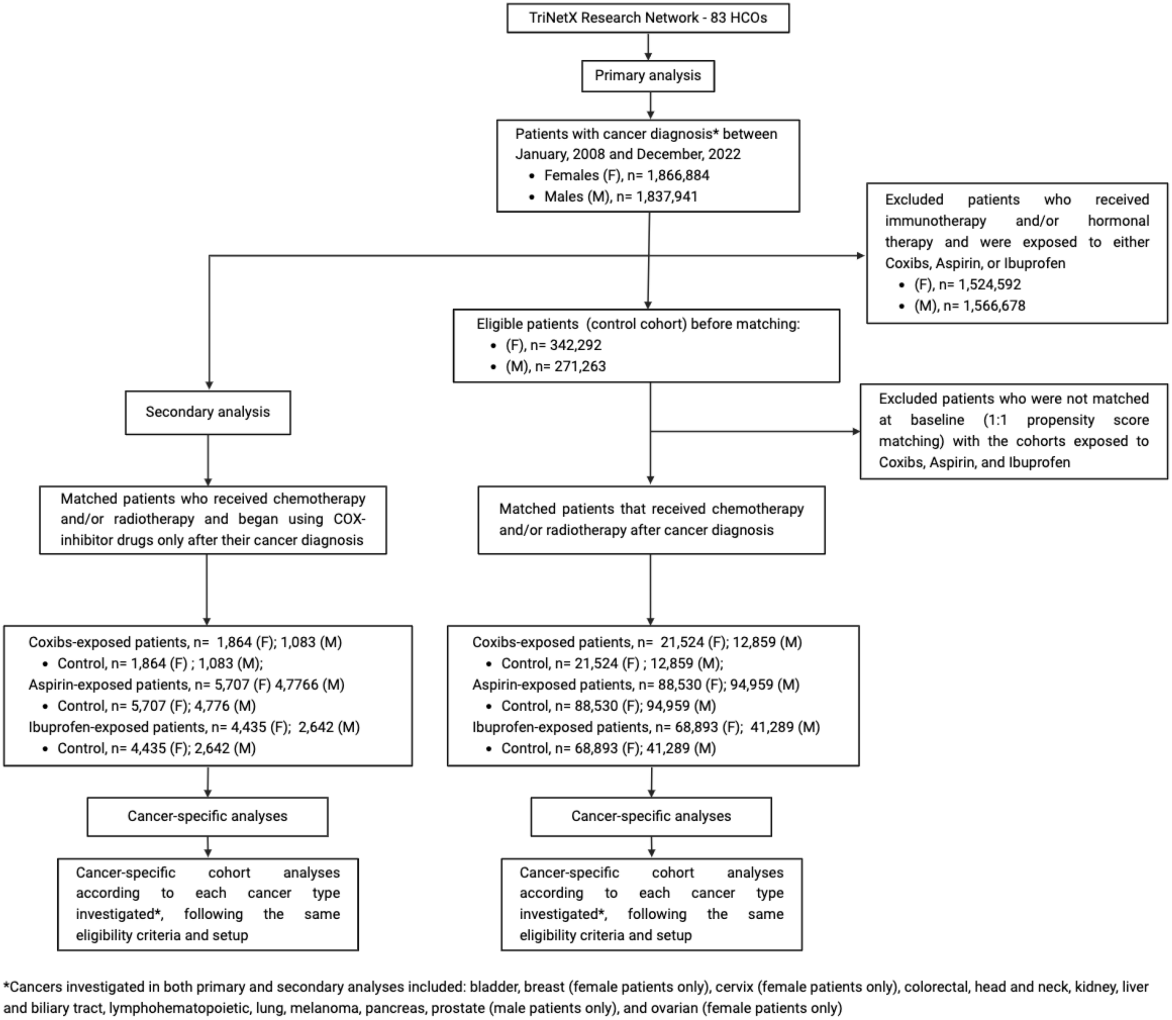
Study flow diagram. The control cohort was formed by patients that had one of the cancers investigated and were treated with chemotherapy and/or radiotherapy without exposure to any of the NSAIDs assed (Coxibs, Aspirin, or Ibuprofen). This control cohort was then compared to NSAIDs exposed cohorts, which were only exposed to either Coxibs, Aspirin, or Ibuprofen during their oncologic treatment with chemotherapy and/or radiotherapy. Cancer-specific analyses using the same eligibility criteria and setup were performed to address the impact of these drugs on specific cancer types. The secondary analysis applied the same method to investigate the post-cancer diagnosis impact of COX-inhibitor drugs.

The study population consisted of individuals on radiotherapy and/or chemotherapy treated for various cancer conditions (e.g., colorectal, liver and intrahepatic bile duct carcinoma, pancreas, lung and bronchus, melanoma, breast, cervical, ovary, prostate, kidney, bladder, head and neck, and lymphohematopoietic cancers) between January 1, 2008 and December 31, 2022, excluding those with the prior history of immunotherapy procedures or hormonal treatment (**Supplementary Table S1**). To account for biological sex differences in cancer outcomes (63, 64), we queried different cohorts including either female or male patients. Breast, cervical, and ovarian cancers were included only in the female cohorts, while prostate cancer was assessed only in the context of male patients. Each patient was attributed to one of four cohorts based on the combination of coxibs, aspirin, and ibuprofen prescriptions (1): the control cohort (without coxibs, aspirin, and ibuprofen) (2), the coxibs cohort (only coxibs, but without aspirin and ibuprofen) (3), the aspirin cohort (only aspirin, but without coxibs and ibuprofen), and (4) the ibuprofen cohort (only ibuprofen, but without coxibs and aspirin). Coxibs drugs (**Supplementary Table S2**), aspirin, ibuprofen medications were identified using normalized name and code sets for medications based on the Prescription for Electronic Drug Information Exchange (RxNorm) and on the Anatomical Therapeutic Chemical (ATC) based on both generic and brand names. In addition, to increase the specificity of our findings, we analyzed individual queries for each cancer type included in our primary analysis, following the same eligibility and index event criteria. To strengthen our analysis, we also performed a secondary investigation restricted to patients who were prescribed coxibs, aspirin, or ibuprofen only after cancer diagnosis (**Figure 1**, Study Flow Diagram).

### 2.2 Outcome measures

The primary outcome measure was 5-year mortality after the initial medication (chemotherapy and/or radiotherapy plus COX-inhibitors). The secondary outcomes focused on the presence of potential side effects associated with coxibs and NSAIDs use (61, 62, 65), including emergency department visits, gastrointestinal ulcers, liver toxicity, cardiovascular and cerebrovascular events, hypertensive events, and kidney damage within 5 years after the medications were administered (**Supplementary Table S3**).

### 2.3 Statistical analyses

Baseline characteristics including patient characteristics (age, race/ethnicity), comorbidities (diabetes, body-mass index (BMI), arthritis, connective tissue diseases, long term use of non-steroidal anti-inflammatories) (66–70), medications (metformin, antihypertensive drugs) (71), and the stage of cancer were accounted for as confounding variables. Given the use of aspirin for secondary prevention of cardiovascular events (72), a history of cardiovascular diseases, events, and procedures, as well as antilipemic agents use was also included in the baseline differences. Although frailty performance and comorbidity scales were not available to be included as covariates in baseline TriNetX analysis, diagnosis related to care provider dependency was included in order to account for potential prescription and outcome bias (see **Supplementary Table S4** for the full list of covariates). The study applied a 1:1 propensity score matching (PSM) technique to balance the baseline characteristics by creating matched pairs of patients with similar propensity scores between two study cohorts. The PSM method was performed using logistic regression and nearest neighbor algorithms with a caliper width of 0.1 pooled standard deviation (SD), assuring that matched pairs having similar baseline characteristics. Cox proportional hazard models were applied to assess the risk of all-cause mortality on cancer patients prescribed coxibs, aspirin, or ibuprofen within 5 years of the initial prescription, compared to patients in the non-NSAIDs cohort. Hazard ratios with 95% confidence intervals (95% CI) for the likelihood of all-cause mortality were calculated and a two-sided *p*<0.05 for statistical significance. Logistic regression model was applied to calculate Odds ratios with 95% confidence intervals (95% CI) to measure the association between COX-inhibitors use and possible complications and toxicity, with a two-sided *p*<0.05 for statistical significance. All data queries and statistical analyses were performed on the TriNetX portal. Detailed diagnosis and laboratory codes for baseline characteristics and outcome measures are available in the **Supplementary Table S5**.

## 3 Results

### 3.1 COX-inhibitors association with overall survival of cancer patients under chemotherapy or radiotherapy

**Figure 2:**
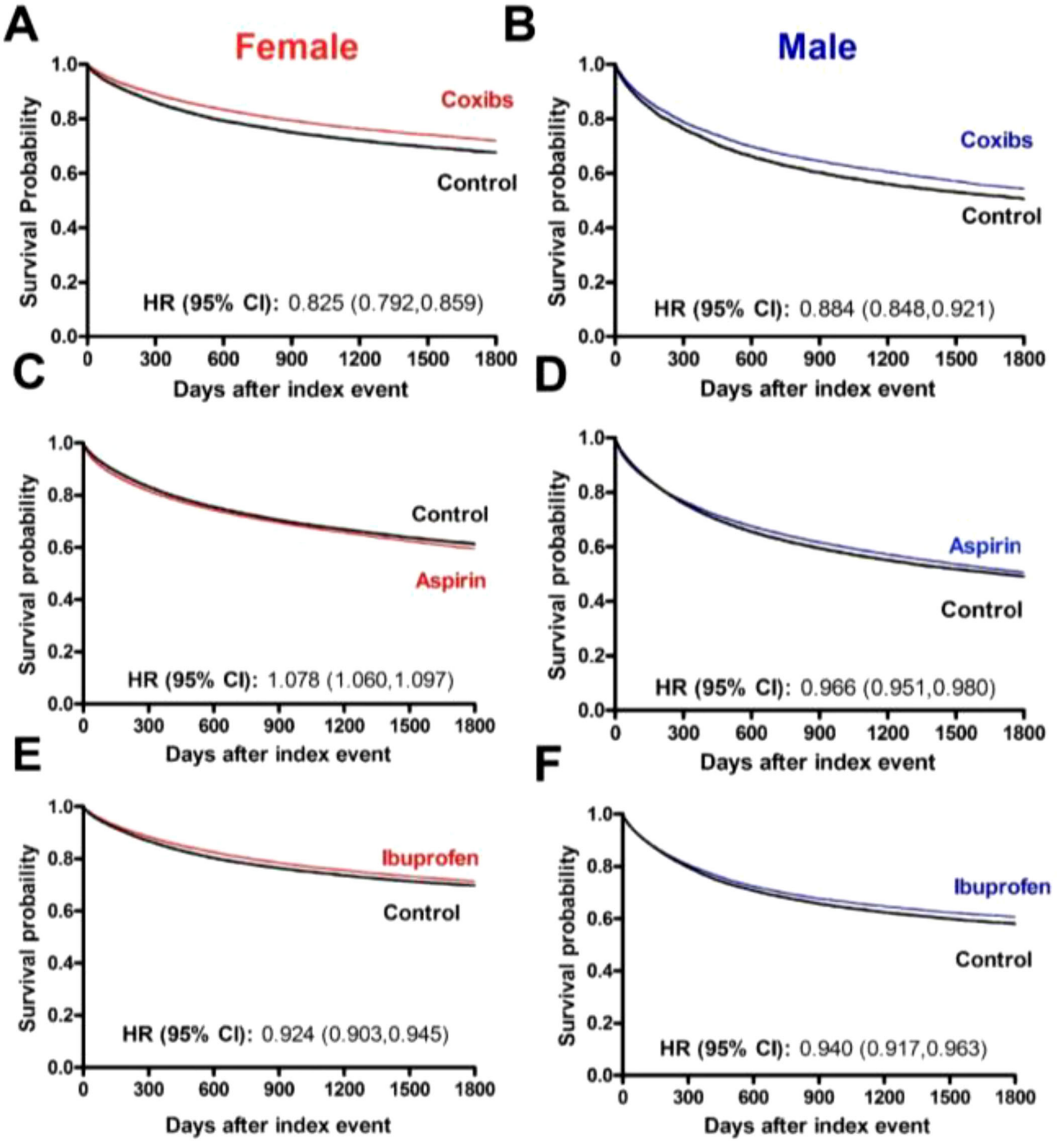
Overall survival of cancer patients that receive COX-inhibitors under chemotherapy and/or radiotherapy. **(A)** Kaplan-Meier curves of female patients and **(B)** male patients that used Coxibs under chemotherapy and/or radiotherapy. The 5-year overall survival rate was, respectively for females and males, 71.53% and 53.93% in the Coxibs group and 67.60% and 50.50% in the control group. **(C)** Kaplan-Meier curves of female patients and **(D)** male patients that used Aspirin under chemotherapy and/or radiotherapy. The 5-year overall survival rate was, respectively for females and males, 59.17% and 50.16% in the Aspirin group and 61.25% and 49.06% in the control group. **(E)** Kaplan-Meier curves of female patients and **(F)** male patients that used Ibuprofen under chemotherapy and/or radiotherapy. The 5-year overall survival rate was, respectively for females and males, 71.05% and 60.35% in the Ibuprofen group and 69.69% and 58.02% in the control group.

Among the cancer types investigated in the female population, we identified 21,524, 88,530 and 68,893 patients who received chemotherapy and/or radiotherapy treatment plus coxibs, aspirin, or ibuprofen, with their respective matched controls. Baseline characteristics of the study and matched control populations are shown in **Table 1**. At the end of the 5-year follow-up period, the use of coxibs (HR, 0.825; 95% CI 0.792-0.859) and ibuprofen (HR, 0.924; 95% CI 0.903-0.945) were associated with significant protection against all-cause mortality. The use of aspirin (HR, 1.078; 95% CI 1.060-1.097), however, was associated with increased risk of mortality (**Figure 2**).

**Table 1:** Propensity score-matched baseline characteristics for female patients treated with radiotherapy and/or chemotherapy.

	With Coxibs (N=21,524)	Without Coxibs (N=21,524)	P-value	With Aspirin (N=88,530)	Without Aspin (N=88,530)	P-value	With Ibuprofen (N=68,893)	Without Ibuprofen (N=68,893)	P-value
**Age at Index, mean +/- SD**	62.7+/-12.8	62.9+/-13.1	0.153	69.0 +/- 11.5	69.4+/-11.4	<0.001	56.5+/-16.6	57.1+/-16.8	<0.001
Race and ethnicity, No. (%)									
**White**	12363 (57.44%)	12298 (57.14%)	0.527	65372 (73.84%)	65885 (74.42%)	0.005	49532 (71.9%)	50421 (73.19%)	<0.001
**Black or African American**	1348 (6.26%)	1376 (6.39%)	0.579	10970 (12.39%)	10856 (12.26%)	0.410	9678 (14.05%)	9694 (14.07%)	0.901
**Hispanic or Latino**	616 (2.86%)	516 (2.40%)	0.003	3475 (3.93%)	3379 (3.82%)	0.237	6246 (9.07%)	6116 (8.88%)	0.220
**Asian**	3928 (18.25%)	3808 (17.69%)	0.132	2637 (2.98%)	2599 (2.94%)	0.594	2901 (4.21%)	2859 (4.15%)	0.572
Diagnosis, No. (%)									
**Long term use of NSAIDs**	670 (3.11%)	600 (2.79%)	0.046	1292 (1.46%)	1318 (1.49%)	0.608	1959 (2.84%)	1806 (2.62%)	0.011
**Family history of arthritis and other diseases of the musculoskeletal system and connective tissue**	183 (0.85%)	141 (0.66%)	0.019	435 (0.49%)	447 (0.51%)	0.685	524 (0.76%)	476 (0.69%)	0.128
**Diseases of the musculoskeletal system and connective tissue**	13435 (62.42%)	13490 (62.67%)	0.584	60415 (68.24%)	62072 (70.11%)	<0.001	43746 (63.5%)	44236 (64.21%)	0.006
**BMI 20-29, adult**	1437 (6.68%)	1530 (7.11%)	0.077	5562 (6.28%)	5589 (6.31%)	0.792	5457 (7.92%)	5389 (7.82%)	0.496
**BMI 30-39, adult**	1801 (8.37%)	1787 (8.30%)	0.807	7198 (8.13%)	7104 (8.02%)	0.412	6948 (10.09%)	7061 (10.25%)	0.314
**BMI 40 or greater, adult**	812 (3.77%)	811 (3.77%)	0.980	3433 (3.88%)	3451 (3.9%)	0.825	3393 (4.93%)	3392 (4.92%)	0.990
**Diabetes mellitus**	3730 (17.33%)	3807 (17.69%)	0.329	23204 (26.21%)	23496 (26.54%)	0.115	10354 (15.03%)	10496 (15.24%)	0.286
**Hypertensive diseases**	9163 (42.57%)	9411 (43.72%)	0.016	55481 (62.67%)	56143 (63.42%)	0.001	28540 (41.43%)	29394 (42.67%)	<0.001
**Diseases of arteries, arterioles and capillaries**	2053 (9.54%)	2035 (9.46%)	0.767	15651 (17.68%)	15471 (17.48%)	0.261	6298 (9.14%)	6538 (9.49%)	0.026
**Ischemic heart diseases**	1993 (9.26%)	1856 (8.62%)	0.021	19946 (22.53%)	18808 (21.25%)	<0.001	4881 (7.09%)	5002 (7.26%)	0.206
**Cerebrovascular diseases**	1373 (6.38%)	1365 (6.34%)	0.874	13175 (14.88%)	12828 (14.49%)	0.020	3606 (5.23%)	3565 (5.18%)	0.619
**Problems related to care provider dependency**	1339 (6.22%)	1276 (5.93%)	0.204	2316 (2.62%)	2324 (2.63%)	0.905	1218 (1.77%)	1058 (1.54%)	0.001
Medications, No. (%)									
**Metformin use**	2108 (9.79%)	2109 (9.80%)	0.987	11303 (12.77%)	11506 (13%)	0.150	5746 (8.34%)	5599 (8.13%)	0.150
**Antihypertensives use**	2869 (13.33%)	3022 (14.04%)	0.032	15230 (17.2%)	14649 (16.55%)	<0.001	8367 (12.15%)	8296 (12.04%)	0.557
**Antilipemic agents**	5658 (26.29%)	5821 (27.04%)	0.076	42302 (47.78%)	41302 (46.65%)	<0.001	16333 (23.71%)	16807 (24.4%)	0.003
Oncology, No. (%)									
**Stage 0**	161 (0.75%)	121 (0.56%)	0.017	681 (0.77%)	638 (0.72%)	0.235	527 (0.77%)	466 (0.68%)	0.052
**Stage 1**	726 (3.37%)	599 (2.78%)	0.000	3321 (3.75%)	3111 (3.51%)	0.008	2931 (4.25%)	2694 (3.91%)	0.001
**Stage 2**	457 (2.12%)	338 (1.57%)	0.000	2139 (2.42%)	1930 (2.18%)	0.001	2153 (3.13%)	1917 (2.78%)	<0.001
**Stage 3**	451 (2.10%)	359 (1.67%)	0.001	2019 (2.28%)	1857 (2.1%)	0.009	2356 (3.42%)	2158 (3.13%)	0.003
**Stage 4**	301 (1.40%)	258 (1.20%)	0.067	2311 (2.61%)	2119 (2.39%)	0.003	2139 (3.11%)	2037 (2.96%)	0.109
Procedures, No. (%)									
**Endovascular Revascularization (Open or Percutaneous, Transcatheter)**	21 (0.10%)	33 (0.15%)	0.102	447 (0.51%)	342 (0.39%)	<0.001	99 (0.14%)	98 (0.14%)	0.943

SD, standard deviation; NSAIDs, non-steroidal anti-inflammatory drugs; BMI, body-mass index.

Among the cancer types investigated in the male population, we identified 12,859, 94,959 and 41,289 patients who received chemotherapy and/or radiotherapy treatment plus coxibs, aspirin, or ibuprofen. Baseline characteristics of the study and matched control populations are shown in **Table 2**. Similar to the female population, at the end of the 5-year follow-up period, the use of coxibs (HR, 0.884; 95% CI 0.848-0.921) and ibuprofen (HR, 0.940; 95% CI 0.917-0.963) were associated with significant protection against all-cause mortality. Aspirin’s use also contributed to survival of male patients (HR, 0.966; 95% CI 0.951-0.980) (**Figure 2**).

**Table 2:** Propensity score-matched baseline characteristics for male patients treated with radiotherapy and/or chemotherapy.

	With Coxibs (N=12,859)	Without Coxibs (N=12,859)	P-value	With Aspirin (N=94,959)	Without Aspin (N=94,959)	P-value	With Ibuprofen (N=41,289)	Without Ibuprofen (N=41,289)	P-value
**Age at Index, mean +/- SD**	63.7+/-13.2	63.9+/-13.4	0.162	68.3+/-11.2	68.9+/-11.15	<0.001	55.13+/-19.5	55.9+/-19.3	<0.001
Race and ethnicity, No. (%)									
**White**	6338 (49.29%)	6195 (48.18%)	0.074	72979 (76.85%)	73555 (77.46%)	0.002	30372 (73.56%)	31054 (75.21%)	<0.001
**Black or African American**	584 (4.54%)	604 (4.7%)	0.552	8435 (8.88%)	8434 (8.88%)	0.994	4693 (11.37%)	4637 (11.23%)	0.538
**Hispanic or Latino**	381 (2.96%)	345 (2.68%)	0.175	3399 (3.58%)	3242 (3.41%)	0.050	3461 (8.38%)	3329 (8.06%)	0.094
**Asian**	3654 (28.42%)	3658 (28.45%)	0.956	3307 (3.48%)	3159 (3.33%)	0.061	1451 (3.51%)	1346 (3.26%)	0.043
Diagnosis, No. (%)									
**Long term use of NSAIDs**	310 (2.41%)	224 (1.74%)	0.000	1409 (1.48%)	1377 (1.45%)	0.541	967 (2.34%)	896 (2.17%)	0.096
**Family history of arthritis and other diseases of the musculoskeletal system and connective tissue**	38 (0.3%)	34 (0.26%)	0.637	224 (0.24%)	228 (0.24%)	0.851	115 (0.28%)	89 (0.22%)	0.068
**Diseases of the musculoskeletal system and connective tissue**	7725 (60.08%)	7778 (60.49%)	0.499	54614 (57.51%)	56285 (59.27%)	<0.001	24608 (59.6%)	25025 (60.61%)	0.003
**BMI 20-29, adult**	980 (7.62%)	966 (7.51%)	0.741	7555 (7.96%)	7547 (7.95%)	0.946	3671 (8.89%)	3720 (9.01%)	0.550
**BMI 30-39, adult**	850 (6.61%)	813 (6.32%)	0.348	7227 (7.61%)	7272 (7.66%)	0.697	2950 (7.15%)	2922 (7.08%)	0.705
**BMI 40 or greater, adult**	229 (1.78%)	217 (1.69%)	0.567	1930 (2.03%)	1925 (2.03%)	0.935	868 (2.1%)	816 (1.98%)	0.200
**Diabetes mellitus**	3146 (24.47%)	3178 (24.71%)	0.643	26748 (28.17%)	27029 (28.46%)	0.152	6692 (16.21%)	6808 (16.49%)	0.275
**Hypertensive diseases**	6418 (49.91%)	6580 (51.17%)	0.043	60297 (63.5%)	61290 (64.54%)	<0.001	18186 (44.05%)	18718 (45.33%)	0.000
**Diseases of arteries, arterioles and capillaries**	1603 (12.47%)	1650 (12.83%)	0.378	20033 (21.1%)	19668 (20.71%)	0.039	4832 (11.7%)	4863 (11.78%)	0.738
**Ischemic heart diseases**	2001 (15.56%)	1916 (14.9%)	0.140	31515 (33.19%)	30151 (31.75%)	<0.001	4899 (11.87%)	5009 (12.13%)	0.239
**Cerebrovascular diseases**	1126 (8.76%)	1096 (8.52%)	0.506	13781 (14.51%)	13313 (14.02%)	0.002	2823 (6.84%)	2816 (6.82%)	0.923
**Problems related to care provider dependency**	1702 (13.24%)	1636 (12.72%)	0.221	2310 (2.43%)	2278 (2.4%)	0.632	936 (2.27%)	765 (1.85%)	<0.001
Medications, No. (%)									
**Metformin use**	1810 (14.08%)	1783 (13.87%)	0.627	12568 (13.24%)	12659 (13.33%)	0.538	3604 (8.73%)	3604 (8.73%)	1.000
**Antihypertensives use**	2679 (20.83%)	2691 (20.93%)	0.854	22967 (24.19%)	22700 (23.91%)	0.152	9731 (23.57%)	9811 (23.76%)	0.512
**Antilipemic agents**	3898 (30.31%)	3999 (31.1%)	0.172	48469 (51.04%)	47534 (50.06%)	<0.001	11050 (26.76%)	11402 (27.62%)	0.006
Oncology, No. (%)									
**Stage 0**	75 (0.58%)	50 (0.39%)	0.025	557 (0.59%)	560 (0.59%)	0.928	171 (0.41%)	178 (0.43%)	0.707
**Stage 1**	311 (2.42%)	263 2.05%()	0.043	2709 (2.85%)	2440 (2.57%)	<0.001	992 (2.4%)	878 (2.13%)	0.008
**Stage 2**	355 (2.76%)	263 (2.05%)	0.000	2848 (3%)	2501 (2.63%)	<0.001	1209 (2.93%)	1033 (2.5%)	0.000
**Stage 3**	374 (2.91%)	286 (2.22%)	0.001	2580 (2.72%)	2327 (2.45%)	<0.001	1241 (3.01%)	1134 (2.75%)	0.026
**Stage 4**	312 (2.43%)	277 (2.15%)	0.145	3821 (4.02%)	3394 (3.57%)	<0.001	1925 (4.66%)	1836 (4.45%)	0.137
Procedures, No. (%)									
**Endovascular Revascularization (Open or Percutaneous, Transcatheter)**	16 (0.12%)	11 (0.09%)	0.336	631 (0.66%)	500 (0.53%)	<0.001	81 (0.2%)	104 (0.25%)	0.090

SD, standard deviation; NSAIDs, non-steroidal anti-inflammatory drugs; BMI, body-mass index.

Both in the male and female populations, the use of coxibs was associated with an increased risk of gastrointestinal ulcers (OR,1.879; 95% CI 1.696-2.081 in females and OR,1.587; 95% CI 1.430-1.761 in males), and the use of ibuprofen was associated with an increased probability of visiting the emergency department (OR,1.322; 95% CI 1.292-1.353 in females and OR,1.278; 95%CI 1.241-1.316 in males). Aspirin use was associated with an increased risk of gastrointestinal ulcers, kidney damage, hypertensive events, and cardiovascular and cerebrovascular events in both sexes, while coxibs and ibuprofen use were protective against kidney damage, hypertensive events, and cardiovascular and cerebrovascular events. Moreover, coxibs reduced risk of emergency department visits (**Supplementary Table S6**).

In our secondary analysis we found 1,864, 5,707, 4,435 female patients and 1,083, 4,476, 2,642 male patients that only started coxibs, aspirin, or ibuprofen, respectively, after cancer diagnosis (**Supplementary Tables S7, S8**). In combination with chemotherapy and/or radiotherapy, COX-inhibitors use was significantly associated with survival in both genders, with coxibs having a greater impact on reducing death risk rates (HR, 0.303; 95% CI 0.231-0.398 in females and HR, 0.378; 95% CI 0.288-0.496 in males), than aspirin (HR, 0.731; 95% CI 0.659-0.810 in females and HR, 0.680; 95% CI 0.617-0.748 in males) and ibuprofen (HR, 0.558; 95% CI 0.482-0.645 in females and HR, 0.685; 95% CI 0.592,0.793 in males). All drugs investigated showed a similar safety profile, though patients receiving aspirin were at increased risk of cardiovascular and cerebrovascular events (OR,1.21; 95% CI 1.118-1.309 in females and OR, 1.362; 95% CI 1.246-1.488 in males) (**Supplementary Table S9**).

### 3.2 COX-inhibitors are associated specific cancer types overall survival during chemotherapy or radiotherapy

**Figure 3:**
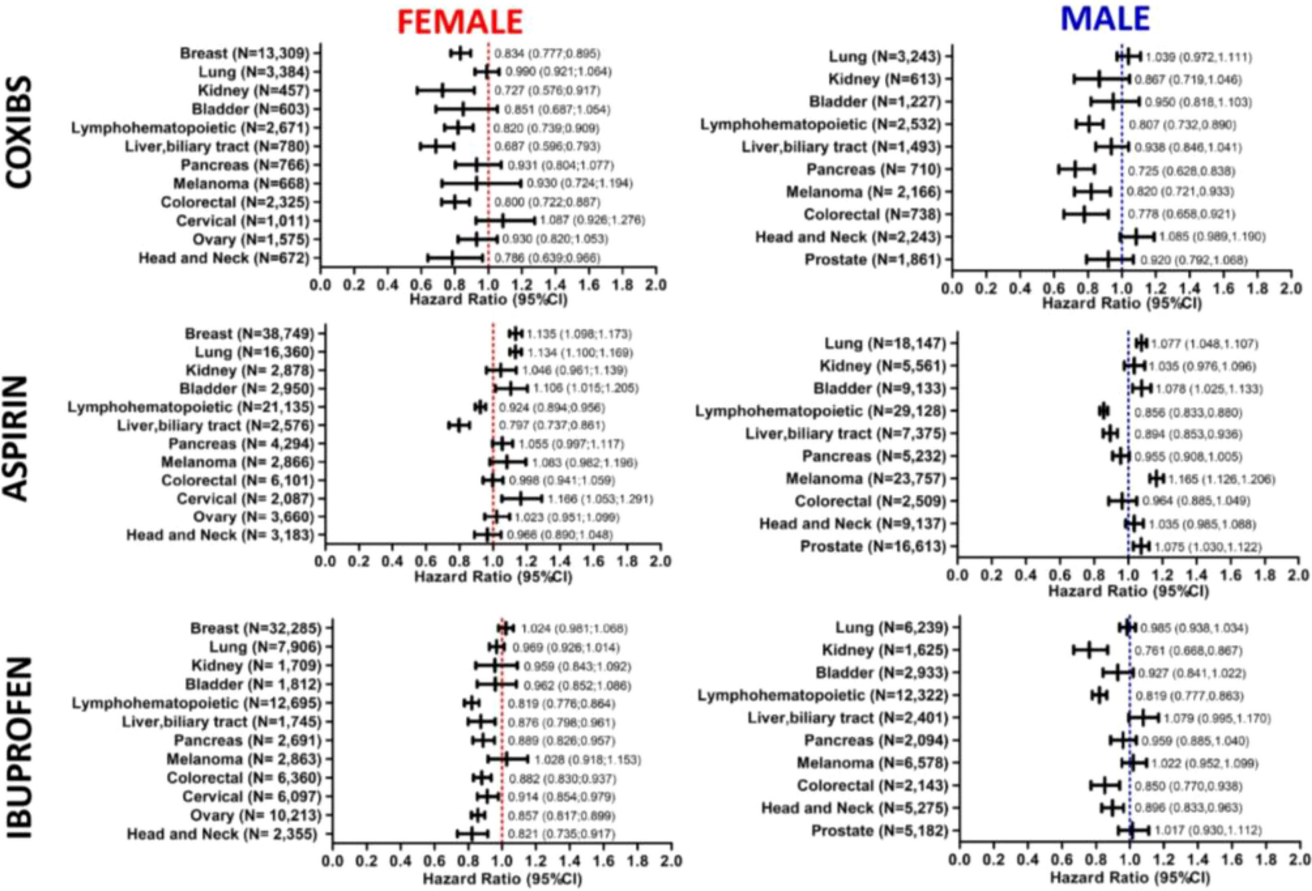
Association of COX-inhibitors with mortality in patients with specific types of cancer. Forest plots demonstrate the hazard ratios (HRs) and 95% confidence intervals (CI) associated with the use of Coxibs, Aspirin and Ibuprofen on mortality of different cancer types in female and male patients. Number of patients included in each cancer type cohort are indicated in the plots.

The use of coxibs correlated significantly with survival in female patients with head and neck (HR,0.786; 95% CI 0.639-0.966), colorectal (HR, 0.800; 95% CI 0.722-0.887), liver and biliary tract (HR, 0.687; 95% CI 0.596-0.793)), lymphohematopoietic (HR, 0.820; 95% CI 0.739-0.909), kidney (HR, 0.727; 95% CI 0.576-0.917), and breast (HR, 0.834; 95% CI 0.777-0.895) cancer. In male patients, coxibs use was significantly associated with protection against mortality in the case of colorectal (HR, 0.778; 95% CI 0.658-0.921), melanoma (HR, 0.82; 95% CI 0.721-0.933), pancreatic (HR, 0.725; 0.628-0.838), and lymphohematopoietic (HR, 0.807; 95% CI 0.732-0.890) cancer (**Figure 3**).

Aspirin use was associated with increased risk of death in patients with cervical (HR,1.166; 95% CI 1.053-1.291), bladder (HR, 1.106; 95% CI 1.015-1.205 in females and HR, 1.078; 95% CI 1.025-1.133 in males), lung (HR, 1.134; 95% CI 1.100-1.169 in females and HR, 1.077; 95% CI 1.048-1.107 in males), breast (HR, 1.135; 95% CI 1.098-1.173), and prostate (HR, 1.075; 95% CI 1.030-1.122) cancers, as well as melanoma male patients (HR, 1.165; 95% CI 1.126-1.206). On the other hand, its use improve survival of patients with lymphohematopoietic cancers (HR,0.924 95% CI 0.894-0.956 in females and HR, 0.856; 95% CI 0.833-0.880 in males), and liver and biliary tract cancers (HR, 0.797; 95% CI 0.737-0.861 in females and HR, 0.894; 95% CI 0.853-0.936 in males) (**Figure 3**).

Ibuprofen use demonstrated an increased association with survival of head and neck (HR,0.821; 95% CI 0.735-0.917 in females and HR, 0.896; 95% CI 0.833-0.963 in males), ovarian (HR, 0.857; 95% CI 0.817-0.899), cervical (HR, 0.914; 95% CI 0.854-0.979), colorectal (HR, 0.882; 95% CI 0.830-0.937 in females and HR 0.850; 95% CI 0.77,0.938 in males), pancreatic (HR, 0.889; 95% CI 0.826-0.957 in females), lymphohematopoietic (HR, 0.819; 95% CI 0.776-0.864 in females and 0.819; HR 0.777-0.863 in males), liver and biliary tract (HR, 0.876; 95% CI 0.798-0.961 in females), and kidney (HR, 0.761; 95% CI 0.668-0.867 in males) cancer patients (**Figure 3**). Patients baseline characteristics are available in the supplement.

Our secondary analysis to assess the benefit of starting COX-inhibitors after cancer diagnosis, though limited by the small number of patients in each cancer subtype cohort, was also able to demonstrate that these drugs, specially coxibs and ibuprofen, are associated with better cancer outcomes. Female patients with colorectal, lung and breast cancer, as well as, male patients with head and neck and lung cancers were benefited by either use of coxibs or ibuprofen. In addition, coxibs reduced death risk of prostate, colorectal, and bladder cancer in male patients. Patients with liver and biliary tract, and lymphohematopoietic cancers generally experienced positive outcomes from generic COX inhibition, with only minor variations observed based on the specific COX inhibitor employed. Data (**Supplementary Tables S10, S11**) and baselines patients characteristics are available in the supplement.

### 3.3 Coxibs use is associated with a safe profile in the majority of the cancer types investigated

Regarding the risk of possible side effects associated with the use of these medications, coxibs use was associated with reduced rates of emergency department visits, and cardiovascular and cerebrovascular events, as well as, was not associated with increased liver toxicity, kidney damage or hypertensive events in the majority of the cancer types investigates.

**Figure 4:**
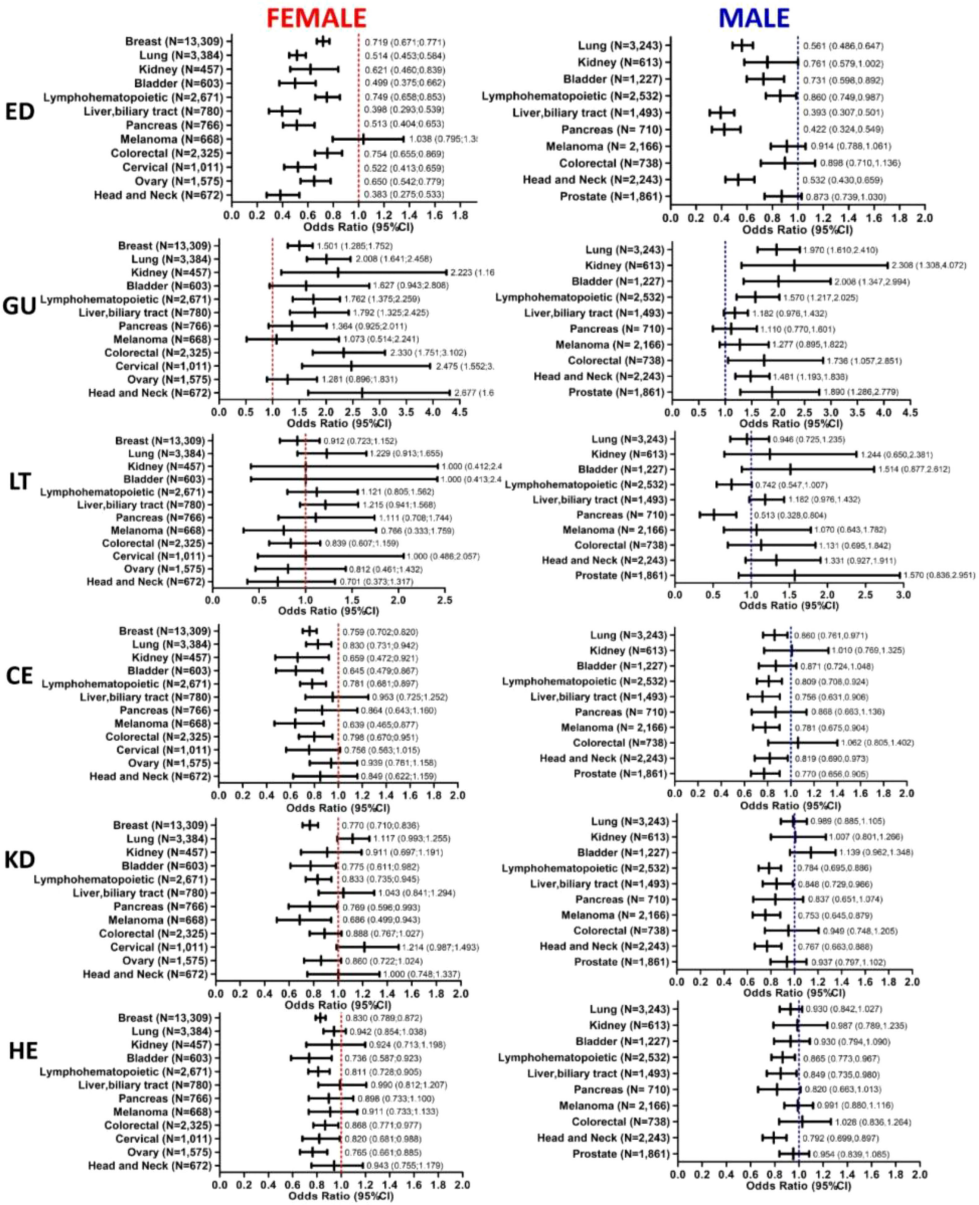
Association of Coxibs plus chemotherapy and/or radiotherapy with medication-related toxic events in cancer patients. Forest plots demonstrate the Odds ratios (ORs) and 95% confidence intervals (CI) of Coxibs association with toxic events incidence on female and male cancer patients, including emergency department visits, gastrointestinal ulcers, liver toxicity, cardiovascular and cerebrovascular events, kidney damage and hypertensive events. Number of patients included in each cancer type cohort are indicated in the plots. DR, Death Risk; ED, Emergency Department visits; GU, Gastrointestinal Ulcers; LT, Liver Toxicity; CE, Cardiovascular and cerebrovascular Events; KD, Kidney Damage; HE, Hypertensive Events.

Coxibs was protective against emergency department visits in several cancer types, including head and neck (OR, 0.383; 95% CI 0.275-0.533 in females and OR, 0.532; 95% CI 0.430-0.659 in males, pancreas (OR, 0.422; 95% CI 0.324-0.549 in males), ovary (OR, 0.650, 95% CI 0.542-0.779), cervical (OR, 0.522; 95% CI 0.413-0.659), colorectal (OR, 0.754; 95% CI 0.655;0.869 in females), liver and biliary tract (OR, 0.398; 95% CI 0.293-0.539 in females and OR, 0.393; 95% CI 0.307-0.501 in males), lymphohematopoietic (OR, 0.749; 95% CI 0.658-0.853 in females and OR, 0.860; 0.749-0.987 in males), bladder (OR, 0.499; 95% CI 0.375-0.662 in females and OR, 0.731; 95% CI 0.598-0.892 in males), kidney (OR, 0.621; 95% CI 0.460;0.839), lung (OR, 0.514; 95% CI 0.453-0.584 in females and OR, 0.561; 95% CI 0.486-0.647 in males) and breast (OR, 0.719; 95% CI 0.671-0.771) cancers. Its use, however, correlated with increased risk of gastrointestinal ulcers in diverse cancer subtypes, both in males and females (**Figure 4**).

On the other hand, use of aspirin and ibuprofen increased risk of emergency department visits in both male and female cancer patients. Moreover, aspirin’s use was associated with cardio and cerebrovascular events as well as kidney damage (**Supplementary Figures S1, S2**).

When only started after cancer diagnosis, these drugs had a similar safety profile between them, protecting cancer patients against emergency department visits, cardio and cerebrovascular events and hypertensive events. Aspirin, however, increased rates of cardiac and cerebrovascular events in ovarian, cervical, liver and biliary tract, and breast cancer in female patients, while increasing risk of the same events in colorectal, bladder, and lung cancers in male patients.

## 4 Discussion

Our findings suggest that selective COX-2 inhibition, represented here by the use of coxibs, is significantly associated with improved cancer outcomes. This includes a reduction in emergency visits and an enhancement in survival, possibly through increased responsiveness to chemotherapy and/or radiotherapy.

During the oncologic treatment, patients commonly present cancer-associated pain demanding the use of pain relief medications. Even though opioids are the primary choice for chronic cancer pain, NSAIDs are often used due to their high efficiency in cancer pain control (73) and, especially, in cases of mild pain (74). Our findings indicate a pattern between the use of NSAIDs, such as aspirin, and an increased risk of emergency department visits and other toxic events. This association may be attributed to the use of aspirin for secondary cardiovascular prevention, which often involves patients with existing cardiovascular comorbidities that could lead to complications and emergencies. Despite controlling for these variables through propensity-score matching and balancing baseline characteristics, a significant difference in cardiovascular comorbidities remained between the aspirin group and the control group (**Tables 1**, **2**). However, restricting the analysis to patients who have only started these drugs after cancer diagnosis did not have the same bias (**Supplementary Tables S7, S8**), but showed similar associations with aspirin and adverse events.

Our data indicate that the COX-inhibitors evaluated here affect patient outcomes differently depending on tumor type and sex. This observation suggests that various tumor sites and types may have differing expression levels and dependencies on the COX pathway (75, 76) and that sex hormones might significantly influence tumor biology and drug response (63, 64). Given the role of precision oncology (77), it is essential to tailor medical decisions to patients’ individualities and tumor profiles. These findings emphasize that even medications used for symptom relief and pain control should be selected with care, as they can directly impact oncological treatment and patient outcomes.

In this context, selective COX-2 inhibitors, such as coxibs, may represent a better choice to control cancer-related pain, as they have been significantly associated with reduced mortality and fewer emergency department visits across various cancer types, including breast, colorectal, melanoma, pancreatic, liver, biliary tract, lymphohematopoietic, lung, and prostate cancers. Based on our secondary analysis, ibuprofen appears to be a reasonable alternative, demonstrating a comparable oncological effect and safety profile to that of COX-2 inhibitors, depending on the type of cancer.

Selective COX-2 inhibitors were developed under the concept of being safer than NSAIDs by not inhibiting COX-1, which spares the gastric mucosa and prevents gastrointestinal ulcer formation. Most COX-2 inhibitors, however, were withdrawn from the market due to their high association with cardiovascular and mortality risks, with only one drug, celecoxib, currently approved for use in the United States (78). Interestingly, our results go in a different direction, suggesting that coxibs use could be associated with an increased risk of gastrointestinal ulcers, without a clear association with higher risks of cardiovascular and hypertensive events, which were increased in the aspirin users cohort. As cancer represents a physiological stress and changes the patient’s homeostatic balance (79), different drug responses between cancer and not-oncologic patients could be a possible explanation for the differences between our results and the classical coxibs-associated side effects, though the limitations and bias inherent to our analysis could also be a reason for that.

Our study had several limitations. Since we used retrospective EHR data, we did not have control over treatment allocation and the results are a consequence of treatment decisions made in the clinic. In addition, HCOs reporting errors can happen, and some patients might have missing information, such as tumor stages. We also did not have information on the duration, dose prescription of the radiotherapy, chemotherapy, coxibs, aspirin, and ibuprofen uses as well as patients’ adherence to those treatments. Aspirin and ibuprofen are medications available over-the-counter and it is possible that patients had taken them without being prescribed, which could influence our results. Furthermore, this study only examined data in a 5-year follow-up and was therefore limited by time period as well as its retrospective nature and inherent biases. On the other hand, a major strength of our study was the relatively large sample of cancer patients that received chemotherapy and/or radiotherapy as well as were prescribed any of the COX-inhibitors analyzed. Moreover, we performed separated analysis based on sex, accounting for the impact that this difference can have on cancer outcomes.

Clinical trials investigating the use of celecoxib, a selective COX-2 inhibitor, as an adjuvant therapy to sensitize chemotherapy effects on patients with breast, lung, colon cancer, and radiotherapy on non-metastatic prostate cancers have demonstrated no evidence of treatment benefit on cancer outcomes as well as no increase in toxic events related to the medication use in breast, lung and prostate cancer patients, while increased hypertension and creatinine elevation risks in colon cancer patients (54, 55, 80, 81). Additionally, a meta-analysis including these and other 9 clinical trials on breast, lung, bladder, colon, gastric, prostate, and ovarian cancer patients found no benefit of celecoxib plus standard chemotherapy on cancer prognosis and identified no associated cardiovascular and gastrointestinal risks, but increased hematologic toxicity associated with this intervention (59). In contrast, there is large evidence from observational and clinical studies supporting the use of coxibs and NSAIDs, such as aspirin, as chemoprevention to reduce risk of cancer, especially colorectal cancer (44, 45, 47–51, 82).

When limiting our analysis to patients who began using COX-inhibitor drugs only after cancer diagnosis, we observed a significant reduction in our sample size, which potentially compromised the power of our analysis. Despite this limitation, a trend favoring the use of these drugs remains apparent, particularly for selective COX-2 inhibition with coxibs in patients with colorectal, liver and biliary tract, lymphohematopoietic and lung cancers, as well as male patients with prostate, and head and neck cancer, and female patients with breast cancer. To validate these findings and confirm their clinical significance, a prospective randomized clinical trial would be necessary.

In the light of that, our results should be carefully interpreted and therefore reinforce the relevance of the COX-2/PGE2 pathway for tumor thriving and cancer therapy resistance, suggesting it as an interesting target for drug repurposing and new cancer drug development. Moreover, this study only examined the benefit of single COX-inhibitors on cancer outcomes, and possible interactions between different NSAIDs and coxibs might reveal different outcomes. In this context, new clinical trials that explore different COX-2 inhibitor drugs, as well as different dosages, treatment durations and combinations, and follow-up periods, are needed to further elucidate the potential benefits of these medications on cancer patients outcomes.

## Data Availability

The data analyzed in this study is subject to the following licenses/restrictions: Data are provided by TriNETX. Requests to access these datasets should be directed to trinetX.com.
