# Supplementary material for: Association of COX-inhibitors with cancer patients’ survival under chemotherapy and radiotherapy regimens: a real-world data retrospective cohort analysis
Source: Front Oncol. 2024 Sep 13;14:1433497. doi: 10.3389/fonc.2024.1433497 (PMC11427433; doi:10.3389/fonc.2024.1433497)
Supplement: Supplementary file 1 [file DataSheet1.pdf]

SUPPLEMENTARY MATERIAL

Figure S1.

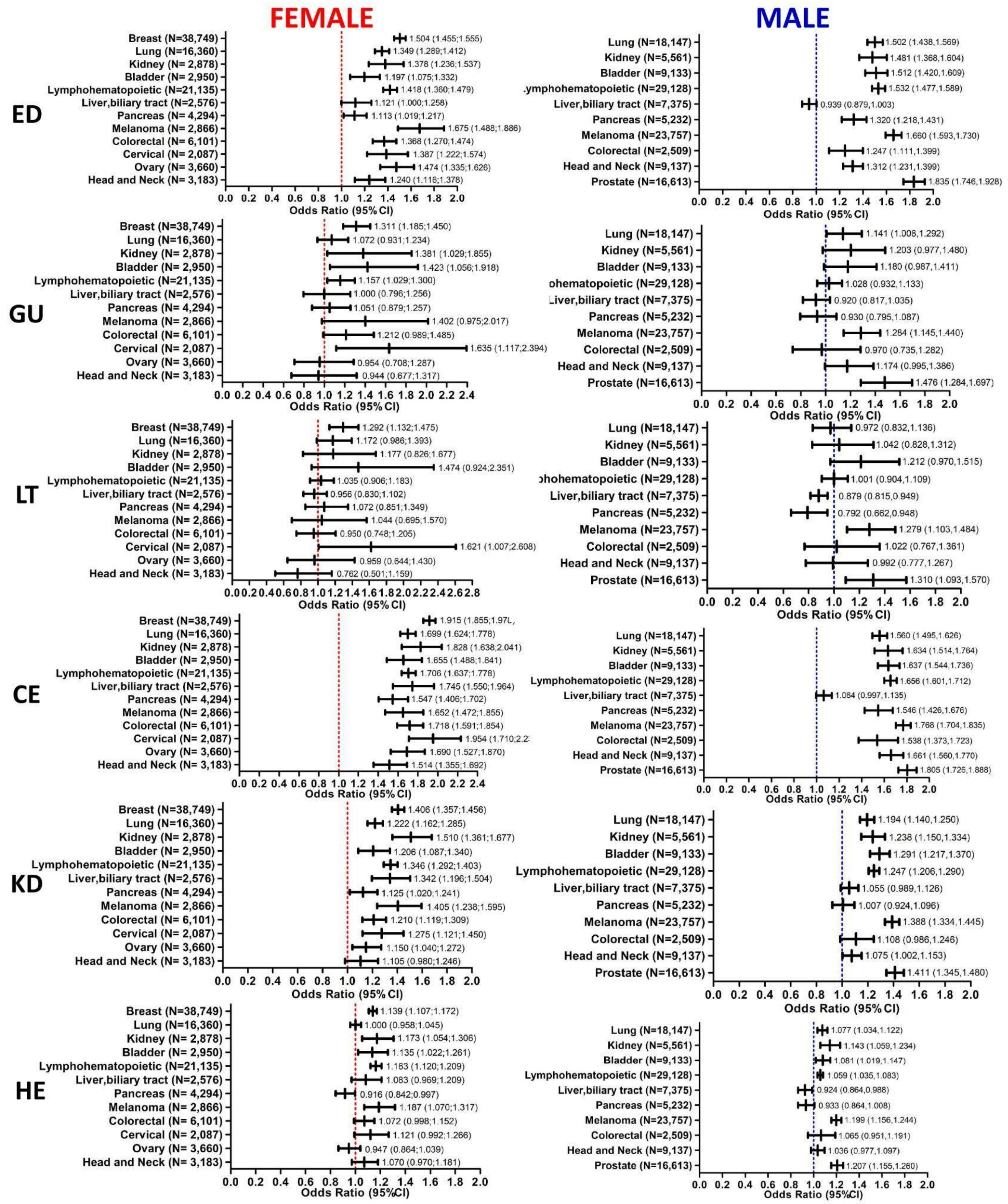

Figure S1. **Association of Aspirin plus chemotherapy and/or radiotherapy with medication-related toxic events in cancer patients.** Forest plots demonstrate the odds ratios (ORs) and 95% confidence intervals (CI) of Aspirin association with toxic events incidence on female and male cancer patients, including emergency department admission, gastrointestinal ulcers, liver toxicity, cardiovascular and cerebrovascular events, kidney damage and hypertensive events. Number of patients included in each cancer type cohort are indicated in the plots. ED, Emergency Department visits; GU, Gastrointestinal Ulcers; LT, Liver Toxicity; CE, Cardiovascular and cerebrovascular Events; KD, Kidney Damage; HE, Hypertensive Events.

Figure S2.

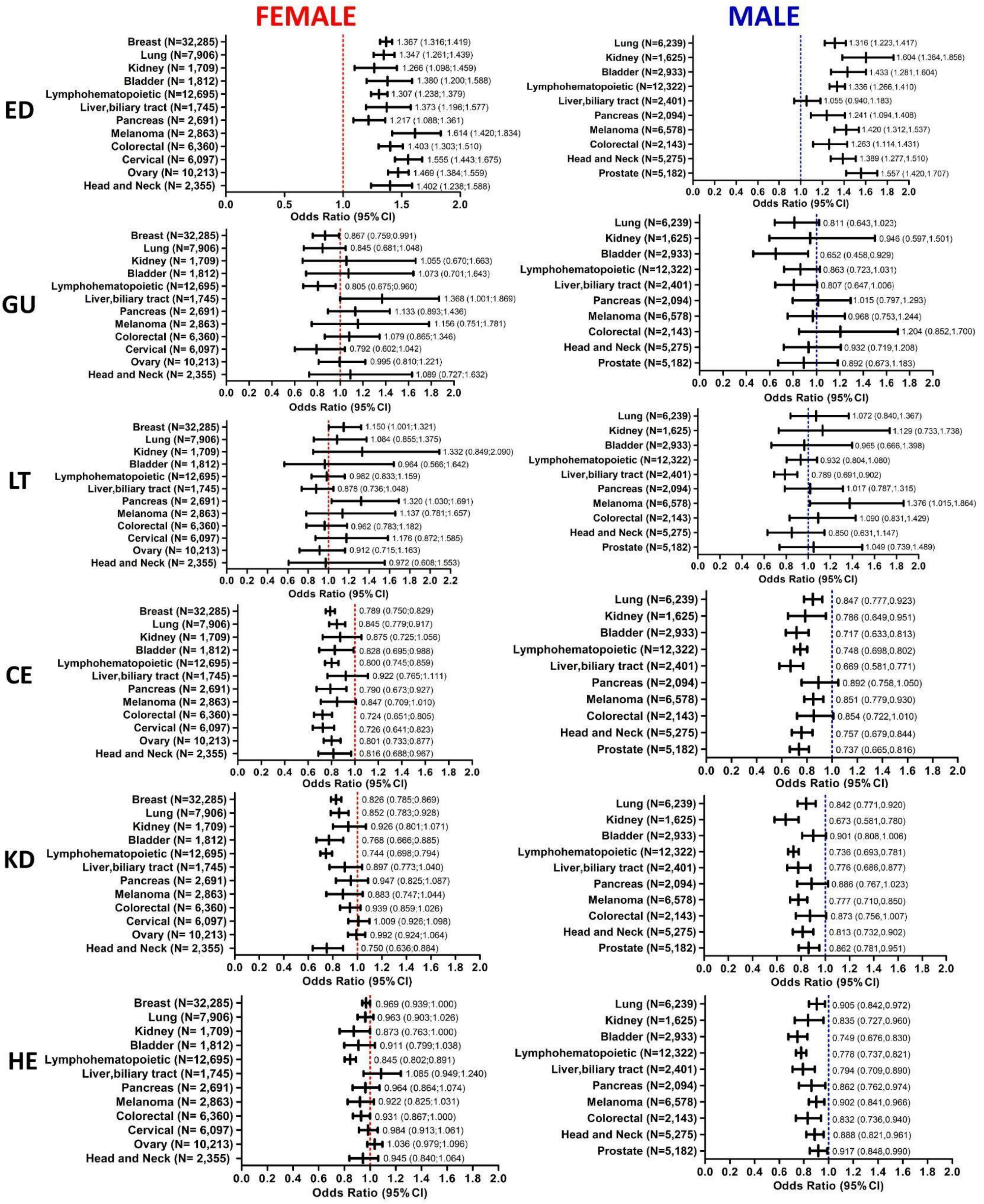
FEMALE
MALE
ED
Breast (N=32,285) 1.367 (1.316;1.419)
Lung (N=7,906) 1.347 (1.261;1.439)
Kidney (N= 1,709) 1.266 (1.098;1.459)
Bladder (N= 1,812) 1.380 (1.200;1.588)
Lymphohematopoietic (N=12,695) 1.307 (1.238;1.379)
Liver,biliary tract (N=1,745) 1.373 (1.196;1.577)
Pancreas (N= 2,691) 1.217 (1.088;1.361)
Melanoma (N= 2,863) 1.614 (1.420;1.834)
Colorectal (N= 6,360) 1.403 (1.303;1.510)
Cervical (N= 6,097) 1.555 (1.443;1.675)
Ovary (N= 10,213) 1.469 (1.384;1.559)
Head and Neck (N= 2,355) 1.402 (1.238;1.588)
Odds Ratio (95% CI)
Lung (N=6,239) 1.316 (1.223,1.417)
Kidney (N=1,625) 1.604 (1.384,1.858)
Bladder (N=2,933) 1.433 (1.281,1.604)
Lymphohematopoietic (N=12,322) 1.336 (1.266,1.410)
Liver,biliary tract (N=2,401) 1.055 (0.940,1.183)
Pancreas (N=2,094) 1.241 (1.094,1.408)
Melanoma (N=6,578) 1.420 (1.312,1.537)
Colorectal (N=2,143) 1.263 (1.114,1.431)
Head and Neck (N=5,275) 1.389 (1.277,1.510)
Prostate (N=5,182) 1.557 (1.420,1.707)
Odds Ratio (95% CI)
GU
Breast (N=32,285) 0.867 (0.759;0.991)
Lung (N=7,906) 0.845 (0.681;1.048)
Kidney (N= 1,709) 1.055 (0.670;1.663)
Bladder (N= 1,812) 1.073 (0.701;1.643)
Lymphohematopoietic (N=12,695) 0.805 (0.675;0.960)
Liver,biliary tract (N=1,745) 1.368 (1.001;1.869)
Pancreas (N= 2,691) 1.133 (0.893;1.436)
Melanoma (N= 2,863) 1.156 (0.751;1.781)
Colorectal (N= 6,360) 1.079 (0.865;1.346)
Cervical (N= 6,097) 0.792 (0.602;1.042)
Ovary (N= 10,213) 0.995 (0.810;1.221)
Head and Neck (N= 2,355) 1.089 (0.727;1.632)
Odds Ratio (95% CI)
Lung (N=6,239) 0.811 (0.643,1.023)
Kidney (N=1,625) 0.946 (0.597,1.501)
Bladder (N=2,933) 0.652 (0.458,0.929)
Lymphohematopoietic (N=12,322) 0.863 (0.723,1.031)
Liver,biliary tract (N=2,401) 0.807 (0.647,1.006)
Pancreas (N=2,094) 1.015 (0.797,1.293)
Melanoma (N=6,578) 0.968 (0.753,1.244)
Colorectal (N=2,143) 1.204 (0.852,1.700)
Head and Neck (N=5,275) 0.932 (0.719,1.208)
Prostate (N=5,182) 0.892 (0.673,1.183)
Odds Ratio (95% CI)
LT
Breast (N=32,285) 1.150 (1.001;1.321)
Lung (N=7,906) 1.084 (0.855;1.375)
Kidney (N= 1,709) 1.332 (0.849;2.090)
Bladder (N= 1,812) 0.964 (0.566;1.642)
Lymphohematopoietic (N=12,695) 0.982 (0.833;1.159)
Liver,biliary tract (N=1,745) 0.878 (0.736;1.048)
Pancreas (N= 2,691) 1.320 (1.030;1.691)
Melanoma (N= 2,863) 1.137 (0.781;1.657)
Colorectal (N= 6,360) 0.962 (0.783;1.182)
Cervical (N= 6,097) 1.176 (0.872;1.585)
Ovary (N= 10,213) 0.912 (0.715;1.163)
Head and Neck (N= 2,355) 0.972 (0.608;1.553)
Odds Ratio (95% CI)
Lung (N=6,239) 1.072 (0.840,1.367)
Kidney (N=1,625) 1.129 (0.733,1.738)
Bladder (N=2,933) 0.965 (0.666,1.398)
Lymphohematopoietic (N=12,322) 0.932 (0.804,1.080)
Liver,biliary tract (N=2,401) 0.789 (0.691,0.902)
Pancreas (N=2,094) 1.017 (0.787,1.315)
Melanoma (N=6,578) 1.376 (1.015,1.864)
Colorectal (N=2,143) 1.090 (0.831,1.429)
Head and Neck (N=5,275) 0.850 (0.631,1.147)
Prostate (N=5,182) 1.049 (0.739,1.489)
Odds Ratio (95% CI)
CE
Breast (N=32,285) 0.789 (0.750;0.829)
Lung (N=7,906) 0.845 (0.779;0.917)
Kidney (N= 1,709) 0.875 (0.725;1.056)
Bladder (N= 1,812) 0.828 (0.695;0.988)
Lymphohematopoietic (N=12,695) 0.800 (0.745;0.859)
Liver,biliary tract (N=1,745) 0.922 (0.765;1.111)
Pancreas (N= 2,691) 0.790 (0.673;0.927)
Melanoma (N= 2,863) 0.847 (0.709;1.010)
Colorectal (N= 6,360) 0.724 (0.651;0.805)
Cervical (N= 6,097) 0.726 (0.641;0.823)
Ovary (N= 10,213) 0.801 (0.733;0.877)
Head and Neck (N= 2,355) 0.816 (0.688;0.967)
Odds Ratio (95% CI)
Lung (N=6,239) 0.847 (0.777,0.923)
Kidney (N=1,625) 0.786 (0.649,0.951)
Bladder (N=2,933) 0.717 (0.633,0.813)
Lymphohematopoietic (N=12,322) 0.748 (0.698,0.802)
Liver,biliary tract (N=2,401) 0.669 (0.581,0.771)
Pancreas (N=2,094) 0.892 (0.758,1.050)
Melanoma (N=6,578) 0.851 (0.779,0.930)
Colorectal (N=2,143) 0.854 (0.722,1.010)
Head and Neck (N=5,275) 0.757 (0.679,0.844)
Prostate (N=5,182) 0.737 (0.665,0.816)
Odds Ratio (95% CI)
KD
Breast (N=32,285) 0.826 (0.785;0.869)
Lung (N=7,906) 0.852 (0.783;0.928)
Kidney (N= 1,709) 0.926 (0.801;1.071)
Bladder (N= 1,812) 0.768 (0.666;0.885)
Lymphohematopoietic (N=12,695) 0.744 (0.698;0.794)
Liver,biliary tract (N=1,745) 0.897 (0.773;1.040)
Pancreas (N= 2,691) 0.947 (0.825;1.087)
Melanoma (N= 2,863) 0.883 (0.747;1.044)
Colorectal (N= 6,360) 0.939 (0.859;1.026)
Cervical (N= 6,097) 1.009 (0.926;1.098)
Ovary (N= 10,213) 0.992 (0.924;1.064)
Head and Neck (N= 2,355) 0.750 (0.636;0.884)
Odds Ratio (95% CI)
Lung (N=6,239) 0.842 (0.771,0.920)
Kidney (N=1,625) 0.673 (0.581,0.780)
Bladder (N=2,933) 0.901 (0.808,1.006)
Lymphohematopoietic (N=12,322) 0.736 (0.693,0.781)
Liver,biliary tract (N=2,401) 0.776 (0.686,0.877)
Pancreas (N=2,094) 0.886 (0.767,1.023)
Melanoma (N=6,578) 0.777 (0.710,0.850)
Colorectal (N=2,143) 0.873 (0.756,1.007)
Head and Neck (N=5,275) 0.813 (0.732,0.902)
Prostate (N=5,182) 0.862 (0.781,0.951)
Odds Ratio (95% CI)
HE
Breast (N=32,285) 0.969 (0.939;1.000)
Lung (N=7,906) 0.963 (0.903;1.026)
Kidney (N= 1,709) 0.873 (0.763;1.000)
Bladder (N= 1,812) 0.911 (0.799;1.038)
Lymphohematopoietic (N=12,695) 0.845 (0.802;0.891)
Liver,biliary tract (N=1,745) 1.085 (0.949;1.240)
Pancreas (N= 2,691) 0.964 (0.864;1.074)
Melanoma (N= 2,863) 0.922 (0.825;1.031)
Colorectal (N= 6,360) 0.931 (0.867;1.000)
Cervical (N= 6,097) 0.984 (0.913;1.061)
Ovary (N= 10,213) 1.036 (0.979;1.096)
Head and Neck (N= 2,355) 0.945 (0.840;1.064)
Odds Ratio (95% CI)
Lung (N=6,239) 0.905 (0.842,0.972)
Kidney (N=1,625) 0.835 (0.727,0.960)
Bladder (N=2,933) 0.749 (0.676,0.830)
Lymphohematopoietic (N=12,322) 0.778 (0.737,0.821)
Liver,biliary tract (N=2,401) 0.794 (0.709,0.890)
Pancreas (N=2,094) 0.862 (0.762,0.974)
Melanoma (N=6,578) 0.902 (0.841,0.966)
Colorectal (N=2,143) 0.832 (0.736,0.940)
Head and Neck (N=5,275) 0.888 (0.821,0.961)
Prostate (N=5,182) 0.917 (0.848,0.990)
Odds Ratio (95% CI)

Figure S2. **Association of Ibuprofen plus chemotherapy and/or radiotherapy with medication-related toxic events in cancer patients.** Forest plots demonstrate the odds ratios (ORs) and 95% confidence intervals (CI) of Ibuprofen association with toxic events incidence on female and male cancer patients, including emergency department admission, gastrointestinal ulcers, liver toxicity, cardiovascular and cerebrovascular events, kidney damage and hypertensive events. Number of patients included in each cancer type cohort are indicated in the plots. ED, Emergency Department visits; GU, Gastrointestinal Ulcers; LT, Liver Toxicity; CE, Cardiovascular and cerebrovascular Events; KD, Kidney Damage; HE, Hypertensive Events.

Table S1.

Code	Definition
**Immunotherapy**	
**ICD-10-PCS: 3E0330M**	Introduction of Antineoplastic, Monoclonal Antibody, into Peripheral Vein, Percutaneous Approach
**ICD-10-PCS: 3E0430M**	Introduction of Antineoplastic, Monoclonal Antibody, into Central Vein, Percutaneous Approach
**ICD-10-PCS: 3E0130M**	Introduction of Antineoplastic, Monoclonal Antibody, into Subcutaneous Tissue, Percutaneous Approach
**ICD-10-PCS: 3E0230M**	Introduction of Antineoplastic, Monoclonal Antibody, into Muscle, Percutaneous Approach
**ICD-10-PCS: 3E0630M**	Introduction of Antineoplastic, Monoclonal Antibody, into Central Artery, Percutaneous Approach
**ICD-10-PCS: 3E0530M**	Introduction of Antineoplastic, Monoclonal Antibody, into Peripheral Artery, Percutaneous Approach
**ICD-10-PCS: 3E00X0M**	Introduction of Antineoplastic, Monoclonal Antibody, into Skin and Mucous Membranes, External Approach
**ICD-10-PCS: 3E04303**	Introduction of Low-dose Interleukin-2 into Central Vein, Percutaneous Approach
**ICD-10-PCS: 3E03303**	Introduction of Low-dose Interleukin-2 into Peripheral Vein, Percutaneous Approach
**ICD-10-PCS: 3E06303**	Introduction of Low-dose Interleukin-2 into Central Artery, Percutaneous Approach
**ICD-10-PCS: 3E05303**	Introduction of Low-dose Interleukin-2 into Peripheral Artery, Percutaneous Approach
**Hormonal therapy**	
**TNX: 1004**	Hormone Therapy

Table S1. Specifications for exclusion criteria due to immunotherapy and hormonal therapy. ICD-10-PCS, ICD-10 procedure coding system. TNX, TriNetX curated code.

Table S2.

ATC code	Name
M01AH01	celecoxib
M01AH02	rofecoxib
M01AH03	valdecoxib
M01AH04	parecoxib
M01AH05	etoricoxib
M01AH06	lumiracoxib
M01AH07	polmacoxib

Table S2. List of Coxibs that patients were exposed to.

Table S3.

Outcomes	Codes	Definition
Death	-	Deceased
Emergency Department	CPT: 1013711	Emergency Department Services
Gastrointestinal ulcers	ICD-10-CM: K25	Gastric ulcer
	ICD-10-CM: K27	Peptic ulcer, site unspecified
	ICD-10-CM: K28	Gastrojejunal ulcer
	ICD-10-CM: K26	Duodenal ulcer
Liver toxicity	ICD-10-CM: K72	Hepatic failure, not elsewhere classified
	ICD-10-CM: K71	Toxic liver disease
Cardiovascular and cerebrovascular events	ICD-10-CM: I20-I25	Ischemic heart diseases
	ICD-10-CM: I50	Heart failure
	ICD-10-CM: I63	Cerebral infarction
Kidney Damage	ICD-10-CM: N17-N19	Acute kidney failure and chronic kidney disease
Hypertensive events	ICD-10-CM: I10	Essential (primary) hypertension
	ICD-10-CM: I11	Hypertensive heart disease
	ICD-10-CM: I16	Hypertensive crisis

Table S3. Specification of the diagnoses and procedures included as toxic events. CPT, current procedural terminology. ICD-10-CM, ICD-10 clinical modification.

Table S4.

List of diagnoses and medications included as covariates	
**Diagnoses**	
**ICD-10 codes**	**Definition**
Z79.1	Long term (current) use of non-steroidal anti-inflammatories (NSAID)
Z82.6	Family history of arthritis and other diseases of the musculoskeletal system and connective tissue
M00-M99	Diseases of the musculoskeletal system and connective tissue
Z68.2	Body mass index [BMI] 20-29, adult
Z68.3	Body mass index [BMI] 30-39, adult
Z68.4	Body mass index [BMI] 40 or greater, adult
E08-E13	Diabetes mellitus
I10-I1A	Hypertensive diseases
I70-I79	Diseases of arteries, arterioles and capillaries
I20-I25	Ischemic heart diseases
I60-I69	Cerebrovascular diseases
Z74	Problems related to care provider dependency
**Medication**	
**Codes**	**Definition**
RxNorm 6809	Metformin
CV490	ANTIHYPERTENSIVES,OTHER
CV350	ANTILIPEMIC AGENTS
**Procedures**	
**Codes**	**Definition**
CPT 1020324	Endovascular Revascularization (Open or Percutaneous, Transcatheter)

Table S4. Specification of diagnoses, medications, and procedures included as covariates. CPT, Current Procedural Terminology

Table S5.

ICD-10 - Code	Diagnosis
C25	Malignant neoplasm of pancreas
C00-C14	Malignant neoplasms of lip, oral cavity and pharynx
C53	Malignant neoplasm of cervix uteri
C56	Malignant neoplasm of ovary
C50	Malignant neoplasm of breast
C67	Malignant neoplasm of bladder
C64	Malignant neoplasm of kidney, except renal pelvis
C22.1	Intrahepatic bile duct carcinoma
C22.0	Liver cell carcinoma
C18	Malignant neoplasm of colon
C19	Malignant neoplasm of rectosigmoid junction
C43	Malignant melanoma of skin
C81-C96	Malignant neoplasms of lymphoid, hematopoietic and related tissue
C34	Malignant neoplasm of bronchus and lung

Table S5. Cancer types included in the analysis according to The International Classification of Disease - Tenth revision (ICD-10).

Table S6.

	Female Patients Under Radiotherapy and/ or Chemotherapy			Male Patients Under Radiotherapy and/ or Chemotherapy		
**OR (95% CI)**	**Coxibs**	**Aspirin**	**Ibuprofen**	**Coxibs**	**Aspirin**	**Ibuprofen**
**Emergency department visit**	0.666 (0.633,0.699)	1.400 (1.371,1.429)	1.322 (1.292,1.353)	0.674 (0.633,0.718)	1.373 (1.346,1.400)	1.278 (1.241,1.316)
**Gastrointestinal ulcers**	1.879 (1.696,2.081)	1.136 (1.07,1.206)	0.923 (0.852,0.999)	1.587 (1.430,1.761)	1.120 (1.064,1.179)	0.992 (0.908,1.083)
**Liver toxicity**	0.954 (0.837,1.088)	1.132 (1.058,1.212)	0.909 (0.842,0.980)	0.928 (0.824,1.045)	1.015 (0.965,1.068)	0.947 (0.88,1.018)
**Cardiovascular and cerebrovascular events**	0.742 (0.701,0.785)	1.819 (1.781,1.857)	0.766 (0.742,0.792)	0.836 (0.785,0.891)	1.737 (1.704,1.770)	0.804 (0.774,0.834)
**Kidney damage**	0.841 (0.798,0.886)	1.370 (1.341,1.400)	0.869 (0.843,0.895)	0.928 (0.877,0.982)	1.326 (1.301,1.353)	0.856 (0.828,0.886)

**Hypertensive events**	0.847 (0.815,0.881)	1.114 (1.094,1.135)	0.671 (0.460,0.980)	0.934 (0.889,0.981)	1.148 (1.127,1.168)	0.867 (0.843,0.892)

Table S6. General association of COX-inhibitors with toxic events in female and male cancer patients.

Table S7.

Propensity Score-Matched Baseline Characteristics for Female Patients Treated with Radiotherapy and/or Chemotherapy									
	With Coxibs (N=1,864)	Without Coxibs (N=1,864)	P-value	With Aspirin (N=5,707)	Without Aspin (N=5,707)	P-value	With Ibuprofen (N=4,435)	Without Ibuprofen (N=4,435)	P-value
**Age at Index, mean +/- SD**	63.8+/-12.2	64.2+/-12.4	0.383	71.2+/-11.6	71.5+/-11.2	0.116	59.7+/-17.0	60.52+/-16.0	0.022
**Race and ethnicity, No. (%)**									
**White**	1139 (61.11%)	1132 (60.73%)	0.814	4333 (75.92%)	4314 (75.59%)	0.678	3173 (71.55%)	3165 (71.36%)	0.851
**Black or African American**	132 (7.08%)	131 (7.03%)	0.949	635 (11.13%)	655 (11.48%)	0.554	607 (13.69%)	669 (15.09%)	0.061
**Hispanic or Latino**	88 (4.72%)	64 (3.43%)	0.047	215 (3.77%)	201 (3.52%)	0.484	393 (8.86%)	398 (8.97%)	0.852
**Asian**	264 (14.16%)	258 (13.84%)	0.777	233 (4.08%)	212 (3.72%)	0.310	189 (4.26%)	174 (3.92%)	0.421
**Diagnosis, No. (%)**									
**Long term use of NSAIDs**	71 (3.81%)	60 (3.22%)	0.328	226 (3.96%)	205 (3.59%)	0.302	160 (3.61%)	138 (3.11%)	0.195
**Family history of arthritis and other diseases of the musculoskeletal system and connective tissue**	24 (1.29%)	13 (0.70%)	0.069	57 (1.00%)	41 (0.72%)	0.105	46 (1.04%)	39 (0.88%)	0.446
**Diseases of the musculoskeletal system and connective tissue**	1348 (72.32%)	1346 (72.21%)	0.942	4544 (79.62%)	4595 (80.52%)	0.232	3275 (73.84%)	3320 (74.86%)	0.274
**BMI 20-29, adult**	224 (12.02%)	234 (12.55%)	0.618	833 (14.60%)	776 (13.60%)	0.125	614 (13.84%)	599 (13.51%)	0.643
**BMI 30-39, adult**	238 (12.77%)	244 (13.09%)	0.770	924 (16.19%)	854 (14.96%)	0.071	665 (14.99%)	689 (15.54%)	0.479
**BMI 40 or greater, adult**	96 (5.15%)	96 (5.15%)	1.000	370 (6.48%)	344 (6.03%)	0.315	250 (5.64%)	226 (5.10%)	0.258
**Diabetes mellitus**	331 (17.76%)	355 (19.05%)	0.310	1634 (28.63%)	1640 (28.74%)	0.901	785 (17.7%)	783 (17.66%)	0.956
**Hypertensive diseases**	865 (46.41%)	900 (48.28%)	0.251	3904 (68.41%)	3959 (69.37%)	0.266	2092 (47.17%)	2159 (48.68%)	0.154
**Diseases of arteries, arterioles and capillaries**	234 (12.55%)	207 (11.11%)	0.171	1419 (24.86%)	1382 (24.22%)	0.421	555 (12.51%)	552 (12.45%)	0.923
**Ischemic heart diseases**	189 (10.14%)	177 (9.50%)	0.509	1659 (29.07%)	1704 (29.86%)	0.356	416 (9.38%)	419 (9.45%)	0.913
**Cerebrovascular diseases**	119 (6.38%)	108 (5.79%)	0.451	1176 (20.61%)	1166 (20.43%)	0.817	295 (6.65%)	266 (6.00%)	0.206
**Problems related to care provider dependency**	108 (5.79%)	112 (6.01%)	0.781	227 (3.98%)	173 (3.03%)	0.006	161 (3.63%)	128 (2.89%)	0.048
**Medications, No. (%)**									
**Metformin use**	201 (10.78%)	212 (11.37%)	0.566	704 (12.34%)	683 (11.97%)	0.547	465 (10.49%)	452 (10.19%)	0.650
**Antihypertensives use**	273 (14.65%)	264 (14.16%)	0.675	1270 (22.25%)	1230 (21.55%)	0.365	670 (15.11%)	626 (14.12%)	0.186

**Antilipemic agents**	640 (34.34%)	669 (35.89%)	0.320	2770 (48.54%)	2795 (48.98%)	0.640	1380 (31.12%)	1426 (32.15%)	0.294
**Oncology, No. (%)**									
**Stage 0**	19 (1.02%)	17 (0.91%)	0.738	55 (0.96%)	49 (0.86%)	0.554	28 (0.63%)	22 (0.50%)	0.395
**Stage 1**	102 (5.47%)	84 (4.51%)	0.176	221 (3.87%)	198 (3.47%)	0.252	190 (4.28%)	177 (3.99%)	0.488
**Stage 2**	48 (2.58%)	35 (1.88%)	0.149	128 (2.24%)	103 (1.81%)	0.097	117 (2.64%)	101 (2.28%)	0.273
**Stage 3**	50 (2.68%)	37 (1.99%)	0.158	126 (2.21%)	107 (1.88%)	0.209	101 (2.28%)	74 (1.67%)	0.039
**Stage 4**	30 (1.61%)	25 (1.34%)	0.497	116 (2.03%)	110 (1.93%)	0.687	72 (1.62%)	52 (1.17%)	0.070
**Procedures, No. (%)**									
**Endovascular Revascularization (Open or Percutaneous, Transcatheter)**	10 (0.54%)	0 (0%)	0.002	41 (0.72%)	31 (0.54%)	0.237	10 (0.23%)	10 (0.23%)	1.000

Table S7. Propensity score-matched baseline characteristics for female patients treated with radiotherapy and/or chemotherapy that only started COX-inhibitors use after cancer diagnosis. SD, standard deviation. NSAIDs, non-steroidal anti-inflammatory drugs. BMI, body-mass index.

Tabel S8.

**Propensity Score-Matched Baseline Characteristics for Male Patients Treated with Radiotherapy and/or Chemotherapy**									
	**With Coxibs (N=1,083)**	**Without Coxibs (N=1,083)**	**P-value**	**With Aspirin (N=4,776)**	**Without Aspin (N=4,776)**	**P-value**	**With Ibuprofen (N=2,642)**	**Without Ibuprofen (N=2,642)**	**P-value**
**Age at Index, mean +/- SD**	63.4+/-13.0	64.2+/-13.0	0.177	70.8+/-10.9	71.5+/-10.5	0.003	58.0+/-20.2	58.9+/-19.3	0.140
**Race and ethnicity, No. (%)**									
**White**	563 (51.99%)	543 (50.14%)	0.390	3614 (75.67%)	3588 (75.13%)	0.537	1932 (73.13%)	1950 (73.81%)	0.575
**Black or African American**	57 (51.99%)	47 (4.34%)	0.315	446 (9.34%)	469 (9.82%)	0.424	282 (10.67%)	307 (11.62%)	0.274
**Hispanic or Latino**	34 (3.14%)	31 (2.86%)	0.706	199 (4.17%)	191 (4.00%)	0.679	234 (8.86%)	232 (8.78%)	0.923
**Asian**	246 (22.72%)	253 (23.36%)	0.721	230 (4.82%)	224 (4.69%)	0.773	121 (4.58%)	97 (3.67%)	0.097
**Diagnosis, No. (%)**									
**Long term use of NSAIDs**	35 (3.23%)	31 (2.86%)	0.617	171 (3.58%)	156 (3.27%)	0.399	75 (2.84%)	58 (2.20%)	0.135
**Family history of arthritis and other diseases of the musculoskeletal system and connective tissue**	10 (0.92%)	10 (0.92%)	1.000	25 (0.52%)	16 (0.34%)	0.159	10 (0.38%)	10 (0.38%)	1.000
**Diseases of the musculoskeletal system and connective tissue**	658 (60.76%)	671 (61.96%)	0.566	3380 (70.77%)	3436 (71.94%)	0.205	1806 (68.36%)	1838 (69.57%)	0.341

**BMI 20-29, adult**	136 (12.56%)	132 (12.19%)	0.794	812 (17.00%)	735 (15.39%)	0.032	492 (18.62%)	504 (19.08%)	0.673
**BMI 30-39, adult**	102 (9.42%)	101 (9.33%)	0.941	717 (15.01%)	696 (14.57%)	0.545	336 (12.72%)	308 (11.66%)	0.239
**BMI 40 or greater, adult**	21 (1.94%)	25 (2.31%)	0.551	148 (3.10%)	139 (2.91%)	0.590	92 (3.48%)	101 (3.82%)	0.509
**Diabetes mellitus**	267 (24.65%)	255 (23.55%)	0.547	1577 (33.02%)	1593 (33.35%)	0.728	1405 (53.18%)	1468 (55.56%)	0.082
**Hypertensive diseases**	551 (50.88%)	571 (52.72%)	0.390	3495 (73.18%)	3564 (74.62%)	0.108	532 (20.14%)	540 (20.44%)	0.784
**Diseases of arteries, arterioles and capillaries**	155 (14.31%)	149 (13.76%)	0.711	1497 (31.34%)	1480 (30.99%)	0.707	489 (18.51%)	480 (18.17%)	0.749
**Ischemic heart diseases**	142 (13.11%)	137 (12.65%)	0.748	2013 (42.15%)	2029 (42.48%)	0.740	411 (15.56%)	418 (15.82%)	0.791
**Cerebrovascular diseases**	92 (8.50%)	87 (8.03%)	0.696	1046 (21.90%)	1001 (20.96%)	0.262	227 (8.59%)	207 (7.84%)	0.316
**Problems related to care provider dependency**	133 (12.28%)	134 (12.37%)	0.948	233 (4.88%)	209 (4.38%)	0.242	128 (4.85%)	108 (4.09%)	0.183
**Medications, No. (%)**									
**Metformin use**	160 (14.77%)	151 (13.94%)	0.581	661 (13.84%)	658 (13.78%)	0.929	279 (10.56%)	276 (10.45%)	0.893
**Antihypertensives use**	239 (22.07%)	216 (19.95%)	0.225	1558 (32.62%)	1545 (32.35%)	0.776	786 (29.75%)	784 (29.67%)	0.952
**Antilipemic agents**	381 (35.18%)	353 (32.60%)	0.204	2607 (54.59%)	2584 (54.10%)	0.637	872 (33.01%)	873 (33.04%)	0.977
**Oncology, No. (%)**									
**Stage 0**	10 (0.92%)	12 (1.11%)	0.668	45 (0.94%)	29 (0.61%)	0.062	17 (0.64%)	16 (0.61%)	0.861
**Stage 1**	37 (3.42%)	29 (2.68%)	0.317	177 (3.71%)	142 (2.97%)	0.046	64 (2.42%)	55 (2.08%)	0.404
**Stage 2**	30 (2.77%)	24 (2.22%)	0.408	155 (3.25%)	118 (2.47%)	0.023	59 (2.23%)	58 (2.20%)	0.926
**Stage 3**	33 (3.05%)	19 (1.75%)	0.049	138 (2.89%)	122 (2.55%)	0.314	59 (2.23%)	50 (1.89%)	0.384
**Stage 4**	23 (2.12%)	12 (1.11%)	0.061	122 (2.55%)	116 (2.43%)	0.694	72 (2.73%)	57 (2.16%)	0.181
**Procedures, No. (%)**									
**Endovascular Revascularization (Open or Percutaneous, Transcatheter)**	10 (0.92%)	10 (0.92%)	1.000	47 (0.98%)	33 (0.69%)	0.116	10 (0.38%)	10 (0.38%)	1.000

Table S8. Propensity score-matched baseline characteristics for male patients treated with radiotherapy and/or chemotherapy that only started COX-inhibitors use after cancer diagnosis. SD, standard deviation. NSAIDs, non-steroidal anti-inflammatory drugs. BMI, body-mass index.

Table S9.

Patients that only started COX-inhibitors after cancer diagnosis						
	Female Patients Under Radiotherapy and/ or Chemotherapy			Male Patients Under Radiotherapy and/ or Chemotherapy		
**HR or OR (95% CI)**	**Coxibs**	**Aspirin**	**Ibuprofen**	**Coxibs**	**Aspirin**	**Ibuprofen**
**Death Risk**	0.303 (0.231,0.398)	0.731 (0.659,0.810)	0.558 (0.482,0.645)	0.378 (0.288,0.496)	0.680 (0.617,0.748)	0.685 (0.592,0.793)
**Emergency Department**	0.363 (0.299,0.440)	0.796 (0.732,0.867)	0.677 (0.611,0.751)	0.453 (0.358,0.572)	0.909 (0.823,1.003)	0.656 (0.579,0.744)
**Gastrointestinal Ulcers**	0.674 (0.459,0.990)	0.606 (0.468,0.785)	0.384 (0.256,0.575)	0.827 (0.539,1.269)	0.664 (0.507,0.871)	0.376 (0.251,0.564)
**Liver Toxicity**	0.228 (0.114,0.456)	0.434 (0.310,0.606)	0.334 (0.228,0.489)	0.268 (0.150,0.477)	0.734 (0.560,0.962)	0.330 (0.226,0.481)
**Cardiovascular and cerebrovascular events**	0.338 (0.273,0.418)	1.210 (1.118,1.309)	0.422 (0.368,0.484)	0.470 (0.364,0.606)	1.362 (1.246,1.488)	0.459 (0.393,0.536)
**Kidney Damage**	0.385 (0.315,0.472)	0.674 (0.617,0.736)	0.413 (0.363,0.471)	0.553 (0.446,0.686)	0.880 (0.801,0.967)	0.421 (0.365,0.485)
**Hypertensive events**	0.497 (0.434,0.570)	0.610 (0.566,0.657)	0.509 (0.466,0.556)	0.565 (0.475,0.674)	0.784 (0.718,0.855)	0.507 (0.453,0.568)

Table S9. General association of COX-inhibitors with mortality and toxic events in female and male cancer patients that only started COX-inhibitors use after cancer diagnosis (secondary analysis).

Table S10.

Female patients that only started COX-inhibitors after cancer diagnosis							
Coxibs							
	**DR**	**ED**	**GU**	**LT**	**CE**	**KD**	**HE**
**Head and Neck (N= 52)**	0.208 (0.025;1.695)	0.646 (0.257;1.626)	(-,-)	1.000 (0.377;2.652)	1.000 (0.377;2.652)	(-,-)	0.450 (0.184;1.101)
**Ovary (N= 101)**	0.512 (0.200;1.310)	0.622 (0.282;1.369)	(-,-)	(-,-)	0.716 (0.320;1.603)	0.681 (0.314;1.477)	0.417 (0.223;0.780)
**Cervical (N= 57)**	0.570 (0.199;1.629)	0.720 (0.287;1.809)	1.000 (0.381;2.626)	1.000 (0.381;2.626)	0.798 (0.314;2.029)	0.695 (0.300;1.610)	0.321 (0.146;0.705)
**Colorectal (N= 182)**	0.440 (0.224;0.864)	0.306 (0.170;0.552)	1.214 (0.511;2.885)	1.000 (0.406;2.464)	0.445 (0.210;0.942)	0.342 (0.184;0.637)	0.517 (0.333;0.801)
**Melanoma (N= 72)**	0.225 (0.050;1.019)	0.522 (0.221;1.235)	(-,-)	(-,-)	0.732 (0.298;1.797)	1.000 (0.389;2.572)	0.696 (0.351;1.379)
**Pancreas (N= 36)**	0.454 (0.171;1.205)	0.769 (0.281;2.104)	(-,-)	1.000 (0.357;2.805)	1.000 (0.357;2.805)	1.000 (0.357;2.805)	0.400 (0.154;1.040)
**Liver and biliary tract (N= 46)**	0.218 (0.049;0.960)	0.787 (0.301;2.058)	1.000 (0.371;2.693)	1.000 (0.371;2.693)	1.000 (0.371;2.693)	0.474 (0.189;1.191)	0.331 (0.133;0.821)
**Lymphohematopoietic (N= 245)**	0.284 (0.143;0.565)	0.403 (0.250;0.650)	0.826 (0.350;1.950)	1.000 (0.409;2.447)	0.296 (0.173;0.504)	0.292 (0.180;0.474)	0.378 (0.260;0.549)

**Bladder (N= 53)**	0.453 (0.146;1.406)	0.716 (0.282;1.814)	(-,-)	(-,-)	1.000 (0.378;2.646)	0.384 (0.158;0.929)	0.255 (0.112;0.578)
**Kidney (N=29)**	0.334 (0.070;1.578)	1.000 (0.339;2.953)	(-,-)	(-,-)	1.000 (0.339;2.953)	0.491 (0.171;1.413)	0.428 (0.148;1.233)
**Lung (N= 200)**	0.286 (0.153;0.534)	0.300 (0.163;0.551)	1.000 (0.423;2.363)	(-,-)	0.594 (0.330;1.067)	0.383 (0.217;0.676)	0.484 (0.314;0.744)
**Breast (N= 1,252)**	0.275 (0.163;0.463)	0.372 (0.284;0.486)	0.804 (0.450;1.437)	0.712 (0.315;1.609)	0.392 (0.295;0.521)	0.395 (0.295;0.530)	0.481 (0.406;0.570)
**Aspirin**							
	**DR**	**ED**	**GU**	**LT**	**CE**	**KD**	**HE**
**Head and Neck (N= 140)**	0.306 (0.153;0.613)	0.711 (0.425;1.188)	1.000 (0.403;2.483)	1.000 (0.403;2.483)	1.351 (0.813;2.246)	0.955 (0.525;1.736)	0.892 (0.558;1.426)
**Ovary (N= 165)**	0.909 (0.566;1.460)	0.780 (0.478;1.273)	1.000 (0.405;2.470)	1.000 (0.405;2.470)	1.774 (1.104;2.849)	0.528 (0.320;0.873)	0.516 (0.333;0.800)
**Cervical (N= 100)**	1.159 (0.547;2.457)	0.516 (0.274;0.973)	1.000 (0.397;2.519)	(-,-)	2.504 (1.315;4.766)	0.850 (0.446;1.621)	0.568 (0.324;0.995)
**Colorectal (N= 314)**	0.673 (0.441;1.026)	0.763 (0.538;1.083)	1.000 (0.410;2.437)	1.000 (0.410;2.437)	1.228 (0.877;1.720)	0.635 (0.442;0.912)	0.599 (0.437;0.821)
**Melanoma (N= 187)**	1.188 (0.671;2.101)	0.837 (0.496;1.413)	(-,-)	1.000 (0.406;2.462)	0.851 (0.539;1.342)	0.510 (0.307;0.845)	0.446 (0.295;0.676)
**Pancreas (N= 119)**	0.695 (0.438;1.102)	0.825 (0.479;1.422)	0.748 (0.314;1.780)	1.000 (0.400;2.499)	1.096 (0.606;1.981)	0.583 (0.313;1.085)	0.473 (0.282;0.793)
**Liver and biliary tract (N= 108)**	0.555 (0.329;0.938)	0.778 (0.441;1.373)	1.000 (0.398;2.510)	0.745 (0.350;1.586)	1.926 (1.048;3.539)	0.825 (0.478;1.422)	0.963 (0.561;1.652)
**Lymphohematopoietic (N= 1,118)**	0.550 (0.438;0.691)	0.923 (0.763;1.117)	0.551 (0.312;0.972)	0.762 (0.368;1.576)	1.016 (0.852;1.213)	0.614 (0.507;0.743)	0.624 (0.528;0.737)
**Bladder (N= 177)**	0.881 (0.547;1.417)	0.898 (0.570;1.415)	1.000 (0.406;2.466)	1.000 (0.406;2.466)	1.123 (0.736;1.713)	0.629 (0.407;0.972)	0.867 (0.565;1.330)
**Kidney (N= 151)**	1.023 (0.591;1.770)	0.679 (0.412;1.120)	1.000 (0.404;2.477)	1.000 (0.404;2.477)	1.154 (0.722;1.847)	0.875 (0.556;1.376)	0.755 (0.474;1.203)
**Lung (N= 637)**	0.741 (0.592;0.927)	0.850 (0.669;1.079)	1.000 (0.473;2.115)	1.000 (0.413;2.419)	1.208 (0.966;1.511)	0.620 (0.475;0.808)	0.538 (0.431;0.672)
**Breast (N= 2,973)**	1.079 (0.901;1.292)	0.823 (0.729;0.929)	0.602 (0.399;0.908)	0.431 (0.224;0.828)	1.292 (1.158;1.442)	0.722 (0.635;0.820)	0.604 (0.545;0.669)
**Ibuprofen**							
	**DR**	**ED**	**GU**	**LT**	**CE**	**KD**	**HE**
**Head and Neck (N= 119)**	0.478 (0.211;1.085)	0.573 (0.305;1.078)	1.000 (0.400;2.499)	(-,-)	0.536 (0.243;1.182)	0.454 (0.203;1.017)	0.350 (0.202;0.609)
**Ovary (N= 239)**	0.588 (0.360;0.961)	0.848 (0.554;1.298)	(-,-)	(-,-)	0.323 (0.174;0.602)	0.348 (0.211;0.574)	0.519 (0.354;0.762)
**Cervical (N= 185)**	1.535 (0.841;2.802)	0.816 (0.525;1.270)	1.000 (0.406;2.463)	1.000 (0.406;2.463)	0.733 (0.388;1.382)	0.614 (0.364;1.036)	0.592 (0.381;0.920)
**Colorectal (N= 333)**	0.583 (0.382;0.890)	0.540 (0.386;0.755)	1.000 (0.411;2.435)	1.000 (0.411;2.435)	0.319 (0.189;0.539)	0.337 (0.220;0.517)	0.491 (0.358;0.674)
**Melanoma (N= 186)**	0.543 (0.270;1.090)	0.559 (0.307;1.015)	1.000 (0.406;2.462)	1.000 (0.406;2.462)	0.366 (0.170;0.785)	0.350 (0.164;0.748)	0.543 (0.344;0.858)
**Pancreas (N= 97)**	0.658 (0.377;1.148)	0.814 (0.434;1.528)	1.000 (0.396;2.523)	1.000 (0.396;2.523)	0.628 (0.267;1.478)	0.854 (0.391;1.863)	0.551 (0.310;0.981)

**Liver and biliary tract (N= 41)**	0.585 (0.202;1.689)	0.891 (0.348;2.284)	(-,-)	1.000 (0.365;2.740)	1.000 (0.365;2.740)	1.137 (0.421;3.067)	0.741 (0.308;1.783)
**Lymphohematopoietic (N= 843)**	0.246 (0.163;0.372)	0.526 (0.415;0.667)	0.494 (0.230;1.062)	0.349 (0.169;0.724)	0.384 (0.287;0.514)	0.290 (0.220;0.382)	0.451 (0.364;0.559)
**Bladder (N= 115)**	1.131 (0.500;2.559)	1.099 (0.602;2.008)	1.000 (0.400;2.503)	(-,-)	1.061 (0.540;2.085)	0.852 (0.448;1.618)	0.691 (0.405;1.180)
**Kidney (N= 90)**	0.511 (0.213;1.227)	0.667 (0.341;1.304)	(-,-)	(-,-)	0.537 (0.231;1.247)	0.412 (0.206;0.823)	0.547 (0.299;0.998)
**Lung (N= 355)**	0.481 (0.341;0.678)	0.702 (0.510;0.965)	0.829 (0.353;1.943)	1.000 (0.411;2.433)	0.605 (0.421;0.869)	0.406 (0.272;0.605)	0.470 (0.346;0.640)
**Breast (N=2,269)**	0.677 (0.520;0.881)	0.616 (0.528;0.718)	0.314 (0.172;0.574)	0.553 (0.293;1.042)	0.335 (0.272;0.412)	0.387 (0.315;0.475)	0.474 (0.419;0.535)

Table S10. Outcomes for female cancer patients that only started using COX-inhibitors plus chemotherapy and/or radiotherapy after cancer diagnosis. Hazard ratios with 95% confidence intervals are represented for Death Risk (DR) outcome. Odds ratios with 95% confidence intervals are represented for Emergency Department visits (ED); Gastrointestinal Ulcers (GU); Liver Toxicity (LT); Cardiovascular and cerebrovascular Events (CE); Kidney Damage (KD); Hypertensive Events (HE). (-,-): indicates that odds ratios (ORs) were not calculated due to the limited number of events during the assessed time span.

Table S11.

Male patients that only started COX-inhibitors after cancer diagnosis							
**Coxibs**							
	**DR**	**ED**	**GU**	**LT**	**CE**	**KD**	**HE**
**Prostate (N=220)**	0.266 (0.110,0.641)	0.378 (0 .218,0.657)	1.000 (0.408,2.453)	(-,-)	0.528 (0.330,0.845)	0.324 (0.1 88,0.559)	0.598 (0. 41,0.873)
**Head and Neck (N=238)**	0.142 (0.062,0.329)	0.536 (0 .242,1.187)	0.478 (0 .219,1.044)	1.000 (0.408,2.449)	0.446 (0.2 42,0.821)	0.208 (0.1 12,0.386)	0.334 (0. 217,0.515)
**Colorectal (N=181)**	0.461 (0.25 2,0.843)	0.316 (0 .177,0.563)	0.756 (0.323,1.771)	1.000 (0.406,2.464)	0.350 (0.181,0.676)	0.449 (0.2 7,0.747)	0.536 (0. 35,0.82)
**Melanoma (N= 43)**	0.265 (0.05 7,1.226)	0.783 (0 .296,2.069)	(-,-)	(-,-)	0.783 (0.296,2.069)	0.783 (0.2 96,2.069)	0.383 (0. 151,0.969)
**Pancreas (N= 59)**	0.504 (0.208,1.219)	1.000 (0.382,2.616)	1.000 (0.382,2.616)	0.799 (0 .315,2.025)	0.799 (0.3 15,2.025)	0.548 (0.2 25,1.336)	0.626 (0. 287,1.365)
**Liver and biliary tract (N= 95)**	0.146 (0.05 8,0.367)	0.814 (0 .333,1.986)	0.540 (0.233,1.25)	0.441 (0 .194,1.002)	0.898 (0.3 62,2.227)	0.516 (0.2 67,0.999)	0.762 (0. 422,1.376)
**Lymphohematopoietic (N=234)**	0.316 (0.16 2,0.615)	0.474 (0.293,0.768)	0.905 (0 .377,2.174)	1.000 (0.408,2.45)	0.400 (0.237,0.675)	0.458 (0.2 92,0.719)	0.404 (0. 276,0.591)
**Bladder (N= 128)**	0.364 (0.16 5,0.802)	0.402 (0 .215,0.754)	1.000 (0.401,2.492)	1.000 (0.401,2.492)	0.726 (0.3 82,1.382)	0.649 (0.3 82,1.101)	0.371 (0. 224,0.615)
**Kidney (N=57)**	0.550 (0.143,2.112)	1.232 (0.520,2.916)	(-,-)	1.000 (0.381,2.626)	0.545 (0.2 23,1.333)	0.410 (0.19,0.886)	0.457 (0. 216,0.967)
**Lung (N= 187)**	0.216 (0.12 4,0.375)	0.517 (0 .265,1.007)	1.097 (0 .472,2.553)	(-,-)	0.426 (0.2 39,0.761)	0.481 (0.2 83,0.817)	0.641 (0. 414,0.994)
**Aspirin**							
	**DR**	**ED**	**GU**	**LT**	**CE**	**KD**	**HE**
**Prostate (N=1,231)**	0.902 (0.72 8,1.117)	0.884 (0.738,1.059)	0.349 (0 .175,0.698)	0.685 (0 .316,1.481)	1.161 (0.990,1.362)	0.772 (0.650,0.917)	0.554 (0. 472,0.651)
**Head and Neck (N=430)**	1.057 (0.76 9,1.452)	0.877 (0.647,1.187)	0.827 (0.411,1.664)	1.000 (0.412,2.428)	1.325 (0.998,1.759)	0.642 (0.4 62,0.892)	0.596 (0. 455,0.781)
**Colorectal (N=407)**	0.745 (0.53 6,1.036)	0.727 (0 .537,0.983)	0.578 (0.261,1.278)	1.000 (0.412,2.429)	1.434 (1.0 81,1.901)	0.786 (0.5 84,1.058)	0.674 (0. 511,0.888)
**Melanoma (N=283)**	0.683 (0.44 7,1.045)	0.446 (0 .289,0.688)	(-,-)	1.000 (0.41,2.441)	0.985 (0.7 03,1.381)	0.739 (0.510,1.071)	0.653 (0. 469,0.91)
**Pancreas (N=143)**	1.332 (0.93 1,1.906)	0.778 (0.475,1.272)	0.821 (0 .343,1.965)	1.000 (0.403,2.482)	1.279 (0.7 86,2.083)	0.875 (0.528,1.452)	0.638 (0.400,1.017)
**Liver and biliary tract (N=239)**	0.585 (0.39 9,0.857)	0.887 (0 .599,1.313)	1.416 (0 .678,2.959)	0.549 (0 .358,0.841)	1.090 (0.757,1.571)	0.933 (0.647,1.345)	0.918 (0. 639,1.319)
**Lymphohematopoietic (N=236)**	0.747 (0.50 9,1.097)	0.755 (0.510,1.119)	1.866 (0 .843,4.133)	5.179 (2 .538,10.567)	1.054 (0.730,1.521)	0.900 (0.623,1.300)	0.728 (0. 504,1.053)

**Bladder (N=565)**	0.865 (0.642,1.164)	0.804 (0.620,1.042)	0.467 (0.218,1.000)	1.000 (0.413,2.422)	1.294 (1.023,1.637)	0.777 (0.610,0.991)	0.619 (0.489,0.785)
**Kidney (N=279)**	0.928 (0.653,1.318)	0.807 (0.557,1.17)	1.000 (0.410,2.442)	1.000 (0.410,2.442)	1.303 (0.931,1.825)	0.865 (0.620,1.208)	0.605 (0.431,0.849)
**Lung (N=617)**	0.746 (0.612,0.910)	1.468 (1.182,1.823)	0.436 (0.225,0.848)	1.000 (0.430,2.324)	1.351 (1.079,1.692)	0.767 (0.600,0.980)	0.776 (0.620,0.970)
**Ibuprofen**							
	**DR**	**ED**	**GU**	**LT**	**CE**	**KD**	**HE**
**Prostate (N=441)**	0.744 (0.480,1.154)	1.058 (0.761,1.47)	0.907 (0.381,2.158)	1.000 (0.412,2.427)	0.564 (0.389,0.817)	0.454 (0.318,0.647)	0.520 (0.398,0.680)
**Head and Neck (N=270)**	0.620 (0.391,0.985)	0.788 (0.524,1.186)	1.000 (0.409,2.443)	1.000 (0.409,2.443)	0.481 (0.289,0.8)	0.364 (0.216,0.612)	0.403 (0.283,0.574)
**Colorectal (N=313)**	0.743 (0.500,1.106)	0.902 (0.645,1.263)	1.000 (0.41,2.437)	0.906 (0.379,2.165)	0.506 (0.325,0.787)	0.348 (0.234,0.518)	0.578 (0.420,0.795)
**Melanoma (N=161)**	0.807 (0.434,1.501)	0.763 (0.442,1.318)	1.000 (0.405,2.472)	1.000 (0.405,2.472)	0.429 (0.238,0.773)	0.433 (0.237,0.790)	0.560 (0.359,0.872)
**Pancreas (N=83)**	1.167 (0.692,1.97)	0.624 (0.316,1.229)	1.000 (0.393,2.546)	1.000 (0.393,2.546)	0.532 (0.228,1.243)	0.445 (0.213,0.931)	0.670 (0.36,1.249)
**Liver and biliary tract (N=93)**	0.610 (0.325,1.143)	0.710 (0.384,1.313)	1.000 (0.395,2.529)	0.310 (0.140,0.689)	0.853 (0.390,1.866)	0.738 (0.372,1.465)	0.432 (0.235,0.795)
**Lymphohematopoietic (N=862)**	0.606 (0.453,0.810)	0.740 (0.592,0.924)	0.494 (0.230,1.062)	0.337 (0.163,0.696)	0.440 (0.329,0.589)	0.360 (0.283,0.458)	0.400 (0.324,0.494)
**Bladder (N=237)**	0.794 (0.489,1.288)	0.574 (0.378,0.872)	1.000 (0.408,2.449)	(-,-)	0.469 (0.299,0.737)	0.314 (0.207,0.478)	0.319 (0.219,0.466)
**Kidney (N=109)**	0.243 (0.113,0.525)	0.608 (0.326,1.135)	1.000 (0.399,2.509)	(-,-)	0.358 (0.162,0.790)	0.463 (0.258,0.829)	0.472 (0.274,0.813)
**Lung (N=243)**	0.715 (0.513,0.996)	0.982 (0.673,1.432)	1.000 (0.409,2.448)	1.000 (0.409,2.448)	0.641 (0.414,0.995)	0.513 (0.326,0.807)	0.688 (0.479,0.989)

Table S11. Outcomes for female cancer patients that only started using COX-inhibitors plus chemotherapy and/or radiotherapy after cancer diagnosis. Hazard ratios with 95% confidence intervals are represented for Death Risk (DR) outcome. Odds ratios with 95% confidence intervals are represented for Emergency Department visits (ED); Gastrointestinal Ulcers (GU); Liver Toxicity (LT); Cardiovascular and cerebrovascular Events (CE); Kidney Damage (KD); Hypertensive Events (HE). (-,-) indicates that odds ratios (ORs) were not calculated due to the limited number of events during the assessed time span.

Baseline male patients characteristics - Primary Analyses (link for excel file)

Baseline female patients characteristics – Primary Analyses (link for excel file)

Baseline male patients characteristics - Secondary Analyses (link for excel file)

Baseline female patients characteristics - Secondary Analyses (link for excel file)
